# An Optimized Two-Herb Chinese Food as Medicine Formula Reduces Cisplatin-Induced Nephrotoxicity in the Treatment of Lung Cancer in Mice

**DOI:** 10.3389/fphar.2022.827901

**Published:** 2022-03-08

**Authors:** Le Shi, Yang Shu, Xiangdong Hu, Waheed Akram, Jun Wang, Shuang Dong, Biaobiao Luo, Jiuliang Zhang, Sheng Hu, Xiaohua Li, Xuebo Hu

**Affiliations:** ^1^ Laboratory of Natural Medicine and Molecular Engineering, College of Plant Science and Technology, National and Local Joint Engineering Research Center for Medicinal Plant Breeding and Cultivation, Hubei Provincial Engineering Research Center for Medicinal Plants, Innovation Academy of International Traditional Chinese Medicinal Materials, Huazhong Agricultural University, Wuhan, China; ^2^ Institute of Agricultural Sciences, University of the Punjab, Lahore, Pakistan; ^3^ Hubei Cancer Hospital, Wuhan, China; ^4^ College of Food Science and Technology, Huazhong Agricultural University, Wuhan, China

**Keywords:** cancer, cisplatin, inflammation, Chinese herbal medicine, nephrotoxicity

## Abstract

Chemotherapy is considered a most effective way to treat cancer. However, it is very common that chemotherapy causes unbearable mental and physical side effects to cancer patients, which ultimately reduces the patients’ confidence of overcoming diseases and compromises the treatment of chemotherapy. Cisplatin (DDP), a widely used anticancer agent for various types of cancers, also damages nontumor cells and tissues, which are mostly related to the activation of the inflammation pathway. Previously, we have discovered a few rational formulas of food as medicine materials that reduced systemic inflammation in *in vitro* and *in vivo* models. Hence, this study reports the ability of an optimized traditional Chinese anti-inflammatory formulation capable of synergizing the antitumor effect of DDP *in vitro* and *in vivo*. More significantly, by formulation of two anti-inflammatory herbal medicine, the *Chrysanthemum × morifolium* (Ramat.) Hemsl [Asteraceae] and *Lonicera japonica* Thunb [Caprifoliaceae] with a mediator *Glycyrrhiza uralensis* Fisch. ex DC [Fabaceae], a best formula relieved the kidney damage imposed by DDP. Treatments of various combinations of major chemical components of the three herbs also exhibited a similar trend for lowering the DDP-induced nephrotoxicity; however, contrary to that of the formula of herbal extracts, all chemical formulas could not recover the body weight and food intake of the tumor-bearing mice treated by DDP. Our findings suggested that the therapeutic index of DDP-based chemotherapy was able to be improved by minimizing toxicities from the two-herb formula to inhibit the inflammation in mouse tumor models and DDP-induced acute kidney injury mouse models.

## Introduction

Cancer is a major cause of sickness in humans, and cancer incidence overall has been increasing (Chen et al., 2016; Feng et al., 2019). Chemotherapy is considered a most effective approach to treat cancer which is often accompanied by various toxic side effects (Ye et al., 2021). Cisplatin (DDP) is the most commonly used platinum anticancer drug. It was launched in the United States in 1978 and became the first-line platinum drug to treat a variety of malignant tumors (Dasari and Tchounwou, 2014). However, the side effects of DDP reduce the patients’ quality of life and treatment compliance (Breen et al., 2020). DDP binds to DNA to produce Pt/DNA conjugates, thereby destroying the structure of DNA and inhibiting cell proliferation (Baik et al., 2003). The major clinical dose-limiting side effect of DDP is nephrotoxicity that further comprises multiple manifestations. According to an estimate, 20%–30% of patients will have acute kidney injury (Miller et al., 2010). The mechanism of DDP-induced nephrotoxicity was studied for a few decades. In 1971, the first study was carried out to observe DDP-induced nephrotoxicity using animal models. It was seen that DDP exposure in animals caused changes of acute tubular necrosis (Kociba and Sleight, 1971). Renal function damage caused by DDP usually occurs within a few days of DDP administration and was accompanied by increased serum inosinic acid and urea nitrogen concentrations, along with decreased urine output (non-oliguria). Furthermore, urine contains glucose and a small amount of protein. This indicates that the proximal tubular function is damaged, and hypomagnesemia often occurs after repeated use of DDP (Santoso et al., 2003). In addition to the dosage, the adverse effects of DDP also depend upon the frequency of administration, patient’s age, gender, hobbies, hypoglycemia, and renal insufficiency (Scott et al., 1989; de Jongh et al., 2001; Dash et al., 2006). DDP causes nephrotoxicity by multiple mechanisms. Firstly, renal accumulation of DDP is greater and its concentration increases in the peripheral blood depending on the treatment time (Frezza et al., 2010). DDP mainly relies on homotransporters in mammals (Ishida et al., 2010). Copper transporters and organic cation transporters play an important role in cellular transportation of DDP and are closely related to the nephrotoxicity caused by DDP (Iinuma et al., 2002). Secondly, the metabolism of DDP in the kidney leads to a sharp increase in the content of thiols in the kidney, thereby damaging the kidney (Townsend and Hanigan, 2002). The main mechanism of DDP is to bind with the DNA molecules of cancer cells to kill them. It is reported that a small amount of DDP can interact with nuclear DNA after entering the cell. However, most of the DDP molecules in the cell bind to mitochondrial DNA or other targets (Burger et al., 1997). The proximal tubule of the kidney is particularly sensitive to DDP, which is the first to be damaged by DDP (Cullen et al., 2007). The pharmacodynamic mechanism of DDP also occurs in the kidney cells. Unfortunately, these anticancer mechanisms inevitably cause damage to the kidneys (Frezza et al., 2010). Other studies have shown that the increase of reactive oxygen species and the production of pro-inflammatory cytokines seem to play an important role in the DDP-induced nephrotoxicity (Kaeidi et al., 2013; Ju et al., 2014).

The three-herb traditional Chinese medicine is composed of *Chrysanthemum × morifolium* (Ramat.) Hemsl. [Asteraceae] (Ju), *Glycyrrhiza uralensis* Fisch. ex DC. [Fabaceae] (Gan), and *Lonicera japonica* Thunb. [Caprifoliaceae] (Jin) and is widely used to cure inflammation (Huang et al., 2016; Yang et al., 2018; Yuan et al., 2020). Preliminary studies demonstrated the *in-vivo* and *in-vitro* anti-inflammatory activity of aqueous extracts of Ju. However, Ju alone caused obvious weight loss of the mice and it could be recovered by additional Gan extract (Wang et al., 2021). Previously, we established an inflammatory endothelial cell model to screen and quantify a variety of anti-inflammatory Chinese herbal medicines (Zhang et al., 2018). Using this novel cell model, we found that Ju and Jin in association with Gan provided better anti-inflammatory effects along with increased immunity of the body (Wang et al., 2021). As inflammation and tumorigenesis are closely related (Weiss, 2008; Singh et al., 2019), we therefore examined the combined effects of anti-inflammatory and antitumor Chinese herbal formulations in both *in vitro* and *in vivo* conditions in the presence of tumor chemotherapy. Our findings show that Ju and Gan can effectively enhance the inhibitory effect of DDP on different pneumonia cells and alleviate the DDP toxicity in mice.

## Materials and Methods

### Herbal Extraction

The roots of Gan and the flower of Ju were used in this study as herbal material. All herbs used in this study were of Chinese origin and identified by Prof. Mo Wang of the College of Plant Sciences and Technology of Huazhong Agricultural University, Wuhan, China. The standard contents of some compounds in herbal material used in this study were quantified by liquid chromatography (HPLC, Primaide, Hitachi Limited). The methods for detecting Gan and Ju by HPLC are shown in Tables 1,2. The injection volume was 20 μl, and the flow rate was kept at 0.8 ml/min. The C18 column (250 × 4.6 mm, 5.0 µm, Agilent, Santa Clara, CA, USA) was used for the chromatographic separations. The contents of the main components in the medicinal materials and aqueous extract of Gan and Ju are shown in Tables 4,5 and the HPLC profiles are shown in Supplementary Figures S1-S4. Compound standards liquiritin (99.5%), chlorogenic acid (3-CQA) (98%), 3,5-dicaffeoylquinic acid (3,5-diCQA) (98.9%), luteoloside (LUS) (99%), and glycyrrhizic acid ammonium salt (99.3%) were purchased from Chengdu Pusi Biotechnology Co., Ltd.

**TABLE 1:** Methods of HPLC on Gan (*λ* = 237 nm).

Time (min)	Composition of mobile phases	
	A: Acetonitrile (%)	B: 0.05% phosphoric acid solution (%)
0–8	19	81
8–35	19–50	81–50
35–36	50–100	50–0
36–40	100–19	0–81

**TABLE 2:** Methods of HPLC on Ju (*λ* = 348 nm).

Time (min)	Composition of mobile phases	
	A: Acetonitrile (%)	B: 0.05% phosphoric acid solution (%)
0–8	19	81
8–35	19–50	81–50
35–36	50–100	50–0
36–40	100–19	0–81

### Cell Culture

Human lung adenocarcinoma cell line PC-9 was generously provided by Professor Moonsoo Jin of the Weill Cornell Medicine, United States. Human lung adenocarcinoma basal epithelial cell line A549 was provided by Professor Nanshan Zhong of Guangzhou Medical University, Guangzhou, China. Human gastric cancer cell line MGC-803 was obtained from Jiangnan University, and human non-negative breast cancer cell line MCF-7 was obtained from Hubei University of Technology. Human non-small cell lung cancer cells H1299 and mouse lung cancer cells LLC were purchased from Annuol (Beijing) Biotechnology Co., Ltd. Cells were cultured in Dulbecco’s modified eagle medium (DMEM; Gibco, USA) supplemented with 10% fetal bovine serum (FBS; Natocor-Industria Biológica, Córdoba, Argentina) and 1% penicillin/streptomycin (HyClone, Logan, UT, USA).

### MTT Assay

MTT metabolic activity assay was performed to determine cell viability. Cancer cells (1 × 10^4^ cells/well) were cultured in DMEM in a 96-well plate at 37°C and exposed to different concentrations of herbal extract combined with DDP IC50 for 24 h. Cells treated with medium only served as negative control. After removing the supernatant of each well followed by PBS washing, 20 µl of MTT solution (5 mg/ml in PBS) was added in the wells along with the 80-µl culture medium. The cells were then incubated for another 4 h, the resultant formazan crystals were dissolved in dimethyl sulfoxide (100 µl), and OD was measured by a microplate reader (Bio-Rad 680, Hercules, CA, United States) at 570 nm with a reference wavelength of 630 nm. All experiments were performed in triplicate, and the relative cell viability (%) was calculated as a percentage relative to the untreated control.

### Animal Study

C57BL/6 mice (1/2 female and 1/2 male, 6 weeks old), purchased from the Hubei Provincial Center for Disease Control and Prevention, were maintained in the animal facility at Huazhong Agriculture University, Wuhan, China. Standard guidelines (ARRIVE 2020; Huazhong Agriculture University for animal experiments) for laboratory animal care were followed. The study protocol was approved by the Huazhong Agricultural University Animal Care Committee (The approval number is HZAUMO-2018-042).

Mice were randomly divided into different groups as indicated, each composed of six to eight mice. Well-grown and non-contaminated LLC cells were trypsinized and resuspended in DMEM medium, mixed by pipetting to ensure cell density at 4 × 10^7^/ml. The skin disinfection of mice was done with the help of 75% alcohol and after iodine wipes. The cell suspension (0.1 ml) was inoculated with the help of a 1-ml syringe under the left sputum. Afterward, the long 1) and short 2) diameters of tumor were measured by a vernier caliper and volume was calculated using the formula “(V) = ab^2^/2”. The tumors of 40–50 mm^3^ volume were selected for downstream analysis.

### Efficacy Experiment

Before the experiment, mice were adaptively fed for 1 week. Mice were randomly grouped into different subgroups when it was started. Details of mouse treatments are provided in Table 3.

**TABLE 3:** Details of treatments used in this study.

Groups	Details of treatments
G1	200 μl of saline
G2	200 μl DDP (10 mg/kg) solution
G3-A	Ju 200 mg/kg + Gan 400 mg/kg
G3-B	Ju 100 mg/kg + Gan 400 mg/kg
G3-C	Jin 200 mg/kg + Gan 400 mg/kg
G3-D	Jin 100 mg/kg + Gan 400 mg/kg
G4-A	DDP 10 mg/kg + Jun 200 mg/kg + Gan 400 mg/kg
G4-B	DDP 10 mg/kg + Ju 100 mg/kg + Gan 400 mg/kg
G4-C	DDP 10 mg/kg + Jin 200 mg/kg + Gan 400 mg/kg
G4-D	DDP 10 mg/kg + Jin 100 mg/kg + Gan 400 mg/kg

Ju = *Chrysanthemum × morifolium* (Ramat.) Hemsl [Asteraceae], Gan = *Glycyrrhiza uralensis* Fisch. ex DC [Fabaceae], Jin = *Lonicera japonica* Thunb [Caprifoliaceae], DPP, cisplatin.

**TABLE 4:** Compounds content in herbal Gan and Ju.

Materials	Compound	Content (%)			Average value (%)	Standard (%)	*R* ^2^
Gan	Liquiritin	0.63	0.65	0.71	0.66	≥0.50	0.9998
	Glycyrrhizic acid	3.14	3.14	3.33	3.20	≥2.00	0.9998
Ju	Chlorogenic acid	0.99	1.10	1.09	1.06	≥0.20	0.9998
	3,5-Dicaffeoylquinic acid	2.81	2.99	2.27	2.69	≥0.70	0.9993
	Luteoloside	0.24	0.20	0.22	0.22	≥0.08	0.9995

**TABLE 5:** compounds content in the extract powder of Gan and Ju.

Materials	Compound	Content (%)			Average value (%)	*R* ^2^
Gan	Liquiritin	1.44	1.49	1.50	1.48	0.9998
	Glycyrrhizic acid	5.33	5.49	5.53	5.45	0.9998
Ju	Chlorogenic acid	0.79	0.86	0.79	0.81	0.9998
	Luteoloside	0.71	0.71	0.71	0.71	0.9995
	3,5-Dicaffeoylquinic acid	1.37	1.50	1.37	1.41	0.9993

The control (G1) group was injected (i.p.) with 200 μl of normal saline starting from day 0 to day 7. G2 (DDP control) was injected (i.p.) with 200 μl (10 mg/kg) of DDP solution at day 0 and 300 μl of saline solution starting from day 1 to day 7. For all groups of G3 A, B, C, and D, mice were injected (i.p.) with 200 μl (10 mg/kg) of DDP solution at day 0 and 300 μl of saline solution starting from day 1 to day 7. Then, 300 μl of formulations (G3-A, B, C, or D) was intragastrically administered under separate treatments, starting from days 1 to 7 after 30 min of saline solution administration. Similarly, all groups of G4 A, B, C, and D were injected (i.p.) with 200 μl (10 mg/kg) of DDP solution at day 0 only and 300 μl of saline solution starting from day 1 to day 7. Then, 300 μl of formulations (G4-A, B, C, or D) was intragastrically administered under separate treatments, starting from days 1 to 7 after 30 min of saline solution administration.

The weight of the mouse diet was weighed daily using the KFS-C1-type electronic scale from day 1 to day 14. The daily feed intake of the mice was determined as the difference in the weight of the previous day’s grain and the weight of the day’s grain. Mice were weighed and recorded daily using KFS-C1 scales from day 1 to day 14. Mice were sacrificed by a single dose of chloral hydrate (400 mg/kg, i.p.; Aladdin, Shanghai, China) and then with neck dislocation on day 14. The exfoliated tumor mass was recorded using a KFS-C1-type electronic scale.

### Nephrotoxicity Test

The following treatments were included in the nephrotoxicity test: Control, DDP, G3B, and G4B. The DDP group was injected (i.p.) with 200 μl (20 mg/kg) of DDP solution once in Day 1. After 30 min, 300 μl of normal saline was intragastrically administered once a day from days 1 to 4. The single Chinese medicine group was injected (i.p.) with normal saline 200 on the first day. After 30 min, 300 μl of each formula was intragastrically administered once a day from days 1 to 4. All doses ensured the tolerance of the mice, and all experimental groups were tested for 4 days.

### The Hematoxylin and Eosin Stain (H&E) Staining

On the fifth day, the kidneys were separated and fixed in neutral paraformaldehyde fixative (Servicebio, Wuhan, China), dehydrated, and embedded in paraffin and 4-μm-thick sections were made, stained with hematoxylin and eosin (H&E) staining. Observations were made after scanning by a panoramic scanner. The procedure of H&E staining is as follows. 1) The isolated tissue sample was cut into appropriate size tissue pieces and fixed in 4% paraformaldehyde for 2 days. 2) The tissue blocks were removed from the fixative, rinsed with PBS for 1 h, and then dehydrated with ethanol of 70%, 80%, 90%, 95%, 95%, 100%, and 100% each for 30 min followed by two steps of xylene, each for 30 min. 3) The tissue block was immersed completely in paraffin solution at 60°C overnight and then cut into slices with a continuous thickness of 5 μm. The slices were water bathed in 40°C for a few seconds and then stored at 4°C until use. 4) The paraffin section was deparaffinized and rehydrated firstly by two steps of xylene bath, each for 15 min. The slides were then dehydrated by two steps of anhydrous ethanol for 5 min each and then 75% ethanol for another 5 min. The slides were then rinsed in distilled water. 5) The slides were sequentially stained with ematoxylin solution, hematoxylin differentiation solution, and bluing solution each for 3–5 min and washed with tap water after each solution. 6) The slides were sequentially bathed in 85% and 95% ethanol, each for 5 min, and then stained with eosin dye for 5 min. 7) Dehydration and sealing: slices were placed in anhydrous ethanol for three times and then two times of xylene, each for 5 min. The slides were sealed by neutral gum until use.

### Blood Chemistry and Blood Routine

Firstly, the EP tube was infiltrated with 7.5% EDTA·2Na solution, and 40 μl of a 7.5% EDTA·2Na solution was retained per tube. The peripheral blood was sampled from the retro orbits of each mouse on the fifth day posttreatment. Previously prepared anticoagulant tubes were used to collect peripheral blood. Afterward, tubes were centrifuged at 3,000 r/min for 5 min, and the supernatant was discarded. The lower red cell layer was stored at 4°C and used for routine blood tests. All blood tests were performed within 24 h.

### Immunohistochemistry (IHC)

On the fifth day, the kidneys were fixed with tissue fixative for 24 h and submitted to Google Biotech Co., Ltd., for immunohistochemistry (IHC) analysis. Simply, the samples were fixed with neutral paraformaldehyde fixative (Servicebio). The first four steps of IHC were the same as abovementioned with H&E. More steps were as follows. 1) Antigen retrieval: the tissue sections were bathed in citric acid (pH 6.0) for antigen retrieval with gradual heating to boiling and then cooling down to room temperature. The sections were washed in PBS (pH 7.4) for 3 times, 5 min each. 2) Blocking: the sections were placed in 3% hydrogen peroxide and incubated at room temperature in darkness for 25 min. Then, sections were washed in PBS (pH 7.4) for three times, 5 min each time. 3) Serum sealing: the slides were blocked with 3% bovine serum albumin (BSA) for 30 min. 4) Primary antibody incubation: the sealing solution is gently removed and the primary antibody prepared with PBS (pH 7.4) in a certain proportion is added to the sections, and then the sections were placed, incubated overnight at 4°C. 5) Secondary antibody incubation: the sections were washed as above in PBS for 3 times, and the tissues were incubated with a horseradish peroxidase-labeled secondary antibody at room temperature for 50 min. 6) DAB chromogenic reaction: the sections were washed as above in PBS for three times. Diaminobenzidine (DAB) color developing: the slights were then placed in DAB solution. 7) Nucleus counterstaining: the sections were counterstained with hematoxylin stain solution for 3 min and washed with tap water followed by differentiation with a hematoxylin differentiation solution for several seconds. After rinsing with tap water, the slides were treated with hematoxylin returning blue solution followed by rinsing with tap water. 8) Dehydration and mounting: the slides were dehydrated as above in H&E and ready for imaging with a Pannoramic Scanner (Pannoramic DESK, P-MIDI, P250, 3DHISTECH, Budapest, Hungary).

### Inflammatory Factor Test

Similarly for inflammatory factor analysis, the liver and kidney of mice were dissected. A scalpel was used to remove about 0.25 g of the tissue section and transferred into a 1.5-ml centrifuge tube. Afterward, 10% of the protease inhibitor was added in the tube. After tissue lysis, slurry was used for subsequent experiments. The expression level of the MCP-1 inflammatory factor in mouse liver and kidney tissues was detected using a CBA Cytokine Detection Kit (BD Company, Franklin Lakes, NJ, USA).

### Data Analysis

The data were expressed as mean ± standard error of the mean (SEM) of quadruplicate samples. Statistical analysis of data was carried out using GraphPad Prism 6. Unpaired Student’s t-test was used to determine the statistical significance in comparison to matching controls. One-way ANOVA was used to compare mean responses among different treatments, followed by Tukey’s *post-hoc* test to determine statistical significance.

## Results

### DDP IC50 of a Group of Cancer Cells

**FIGURE 1:**
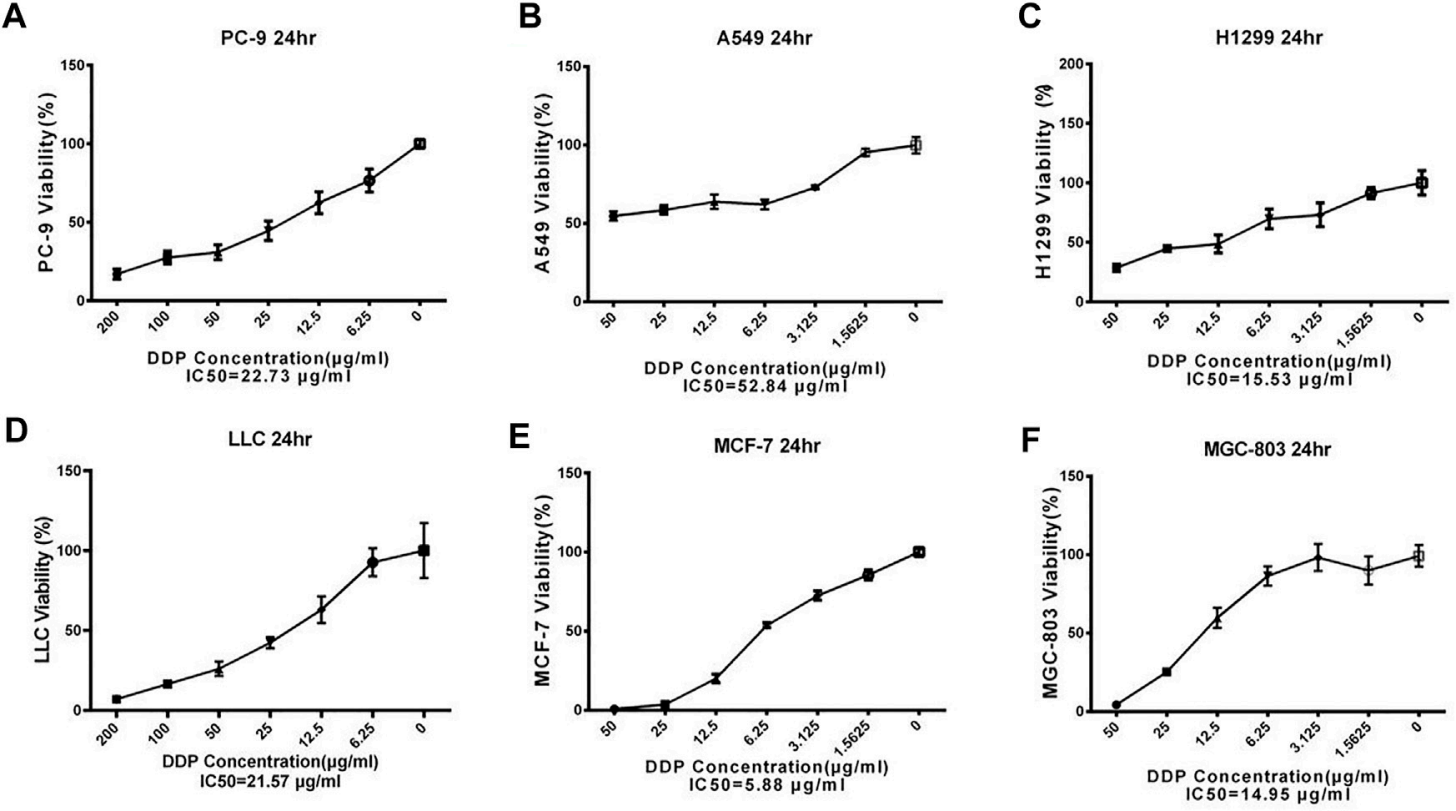
The IC50 of DDP on different cancer cells. Error bars are represented as mean ± SEM. Details of experimental procedures are given in *Materials and Methods*.

MTT results showed varying tolerance levels for DDP and IC50 regarding six gradients of DDP examined for 24 h (Figure 1). The IC50 values of PC-9, A549, H1299, LLC, MGC-803, and MCF-7 were recorded as 22.73, 52.84, 15.53, 57.46, 14.95, and 5.88 μg/ml, respectively. The IC50 values of the obtained cancer cells were helpful to determine the tolerance of different cancer cells against DDP. A549 and LLC showed the strongest tolerance, followed by PC-9, whereas the weakest tolerance was observed for MCF-7 cells. A nearly similar trend was seen for the remaining cancer cells. These IC50 values will be used to formulate treatments for downstream analysis.

### The effect of Single Herbal Extract Combined With DDP on Cancer Cell

**FIGURE 2:**
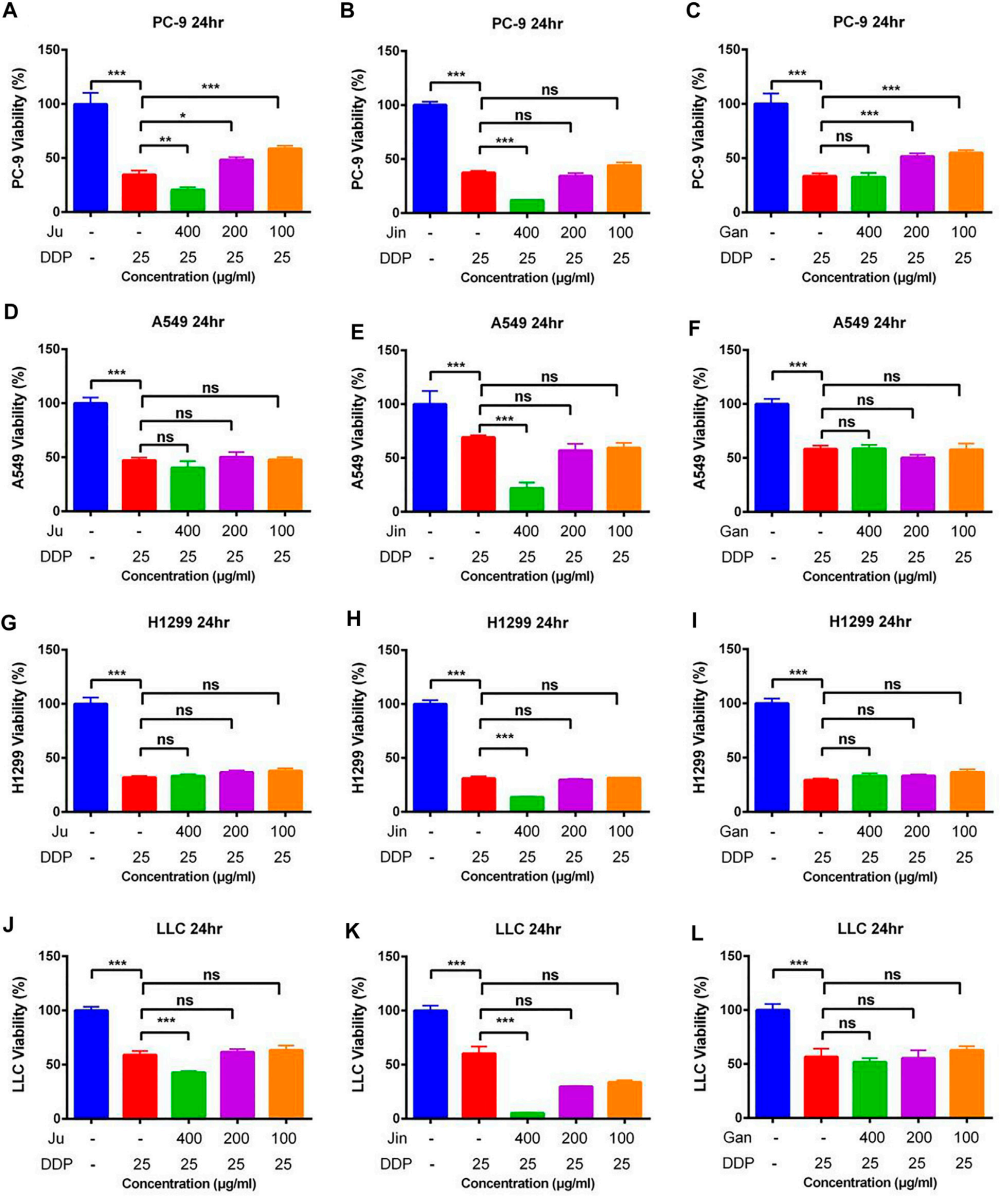
The effect of single Chinese herbal medicine on the antitumor activity of DDP *in vitro*. **(A–C)** Dose of Ju, Jin, or Gan on the antitumor activity of DDP in PC-9 cells. **(D–F)** Dose of Ju, Jin, or Gan on the antitumor activity of DDP in A549 cells. **(G–I)** Dose of Ju, Jin, or Gan on the antitumor activity of DDP in H1299 cells. **(J–L)** Dose of Ju, Jin, or Gan on the antitumor activity of DDP in LLC cells. Error bars are represented as mean ± SEM. *p* values were determined by Student’s t test (*p < .05, **p < .01, ***p < .001).

PC-9 cells are human lung adenocarcinoma cells. Exposure of PC-9 cells with different concentrations of three Chinese herbal formulations in combination with DDP showed variable effects on cell proliferation (Figure 2). Here, Ju aqueous extracts (0.4 mg/ml) significantly increased the inhibitory effect of DDP on PC-9 cell proliferation compared with the DDP-alone (DDP) treatment (Figure 2A) (*p* < 0.01). Similarly, the same dosage of Jin aqueous extracts significantly increased the inhibitory effect of DDP on PC-9 cell proliferation as compared to DDP alone (Figure 2B) (*p* < 0.001), whereas Gan was unable to enhance the cytotoxic effect of DDP (Figure 2C).

In case of A549 (human lung adenocarcinoma basal epithelial) cells, Ju and Gan were unable to enhance the cytotoxic effect when provided in combination with DDP as compared to the DDP-alone treatment (Figures 2D,F). Compared with the DDP group, only 0.4 mg/ml of Jin significantly increased the inhibition of DDP on cell proliferation (Figure 2E) (*p* < 0.001).

Ju and Gan were unable to significantly enhance the cytotoxic effect of DDP in H1299 (human non-small lung cancer) cells as compared to the DDP group (Figures 2G,I) (*p* > 0.05). Here, Jin (0.4 mg/ml) significantly increased the inhibitory effect of DDP on cell proliferation (Figure 2H) (*p* < 0.001).

In case of Lewis lung cancer (LLC) cells, Ju (0.4 mg/ml) significantly increased the inhibitory effect of DDP on cell proliferation as compared to the DDP-alone treatment (Figure 2J) (*p* < 0.001). Similarly, a significant decrease in the cell proliferation was seen when DDP was applied in combination with 0.4, 0.2, or 0.1 mg/ml of Jin (Figure 2K) (*p* < 0.001). Gan was unable to enhance the cytotoxic effects of DDP when applied on LLC cells (Figure 2L) (*p* > 0.05).

### The effect of Aqueous Extracts of Multiple Chinese Herbal formulationnull DDP on Cancer Cells Proliferation

As was observed in a previous experiment, Ju and Jin alleviated sepsis caused by LPS, but the mice infused with Jun or Jin showed significant weight loss. However, the combination of Gan, Jin, and Ju not only maintains/alleviates sepsis but also maintains the weight of mice. However, it is still unknown whether the addition of Gan can promote the cytotoxicity of Ju and Jin in combination with DDP.

**FIGURE 3:**
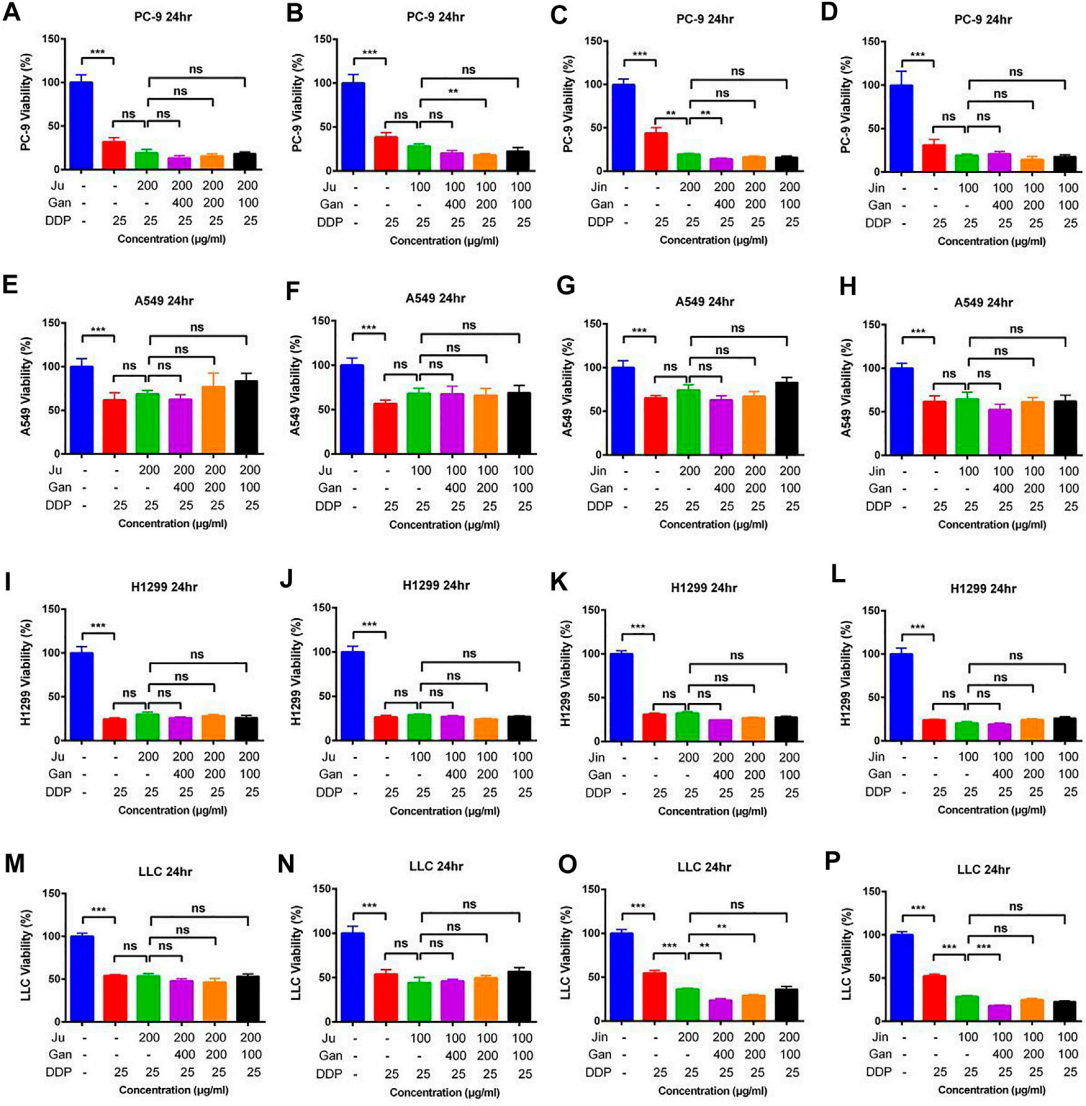
The effect of two Chinese medicine formulas on the antitumor activity of DDP *in vitro* **(A–C)** Dose of Ju + Gan or Jin + Gan on the antitumor activity of DDP in PC-9 cells. **(D–F)** Dose of Ju + Gan or Jin + Gan on the antitumor activity of DDP in A549 cells. (G–I) Dose of Ju + Gan or Jin + Gan on the antitumor activity of DDP in H1299 cells. **(J–L)** Dose of Ju + Gan or Jin + Gan on the antitumor activity of DDP in LLC cells. Error bars are represented as mean ± SEM. *p* values were determined by Student’s t test (*p < .05, **p < .01, ***p < .001).

In case of the PC-9 cell model, G4-B and G4-C treatments significantly increased the cytotoxicity of DDP as compared to the DDP-alone treatment (Figures 3B,C) (*p* < 0.01). In the LLC cell model, only G4-A, G4-B, and G4-C showed the auxiliary effect of Gan (Figures 3O,P) (*p* < 0.01). However, in the H1299 and A549 cell models, Gan was unable to show the auxiliary effect (Figures 3E-L).

### The effect of Chinese Herbal Formulations Combination Extract Combined With DDP on Lung Cancer Tumors in Mice

**FIGURE 4:**
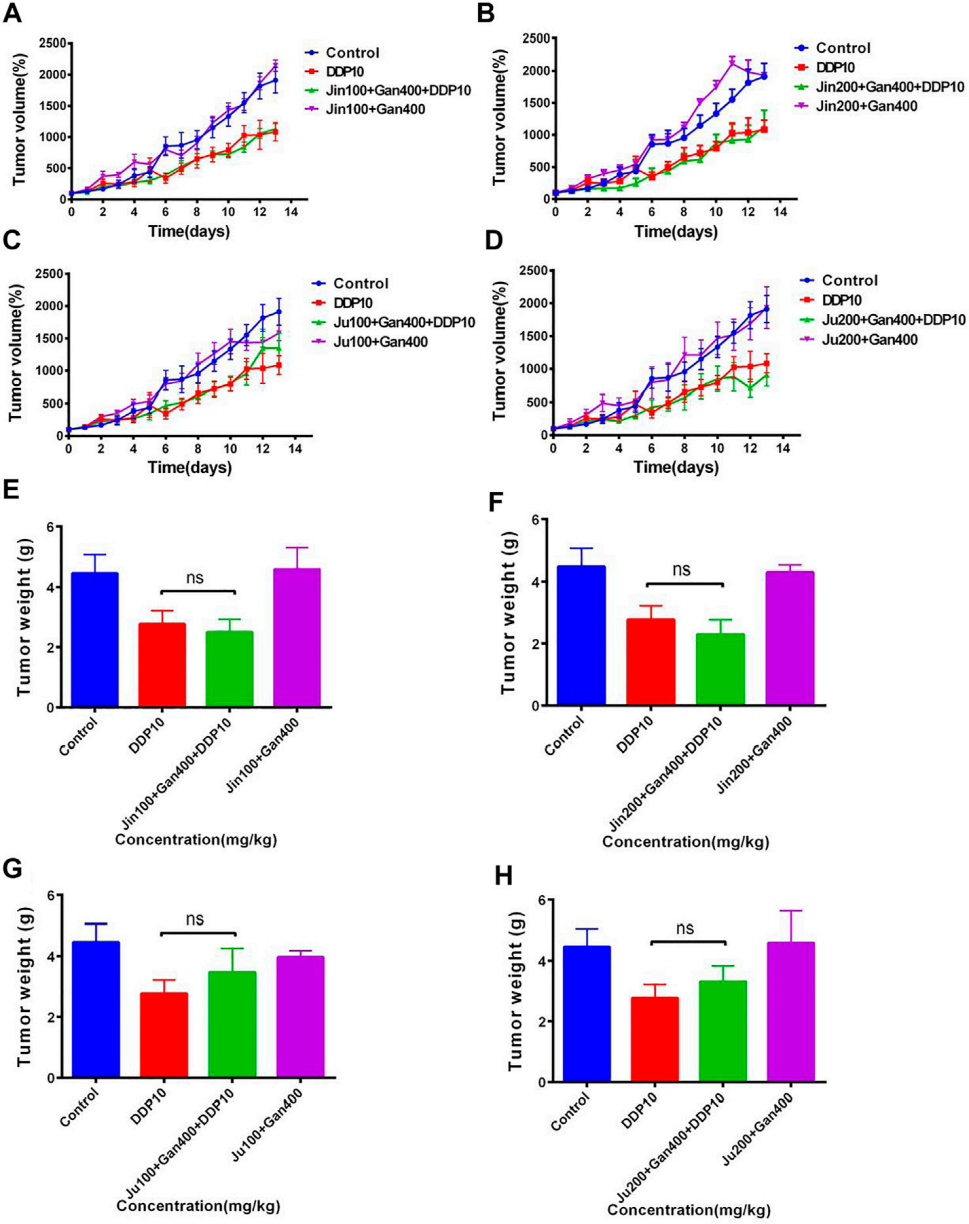
The effect of two Chinese medicine formulas on the antitumor activity of DDP *in vivo*. **(A-B,E-F)** Dose of Ju + Gan on the antitumor activity of DDP in mice. **(C-D,G-H)** Dose of Jin + Gan on the antitumor activity of DDP in mice.

The mouse lung cancer tumor model was established by subcutaneously inoculating LLC cells in C57BL/6 mice. The tumor mass and the average tumor volume of each group of mice were measured on the first day and denoted as 100%. Afterward, the proportion of the average tumor volume of each group was measured in the next 13 days relative to the first day. On the 14th day, the tumor volume of the mice in the model group increased up to 20-fold compared to the first day. Similarly, the tumor volume of the mice in the DDP group increased up to 10-fold as compared to the first day, indicating that the mouse lung cancer tumor model was successfully established and DDP significantly inhibited tumor growth (Figures 4A–D) (*p* < .001).

Individual Chinese herbal formulations (G3-A, G3-B, G3-C, and G3-D) did not exhibit a significant reduction of the tumor growth in mice as compared to the nontreated control mouse group (*p* > .05), whereas Chinese herbal formulations combined with DDP (G4-A, G4-B, G4-C, and G4-D) were unable to significantly reduce the tumor volume compared to the DDP-alone treatment (*p* > .05). At the same time, the weight of the tumor was also measured on the last day of application of Chinese herbal formulation application. No significant difference was seen in tumor weight under the influence of Chinese herbal formulations + DDP as compared to the DDP-alone treatment (Figures 4E,F). (*p* > .05). These results indicate that the Chinese herbal formulations are unable to increase the suppressive effect of DDP on tumor growth.

### Effect of Chinese Medicine Formulas on the Weight Loss and Decline of Food Intake in Mice Induced by DDP

Findings showed that the Chinese herbal formulations did not increase the antitumor effect of DDP. The body weight and food intake of each mouse were monitored up till 14 days.

**FIGURE 5:**
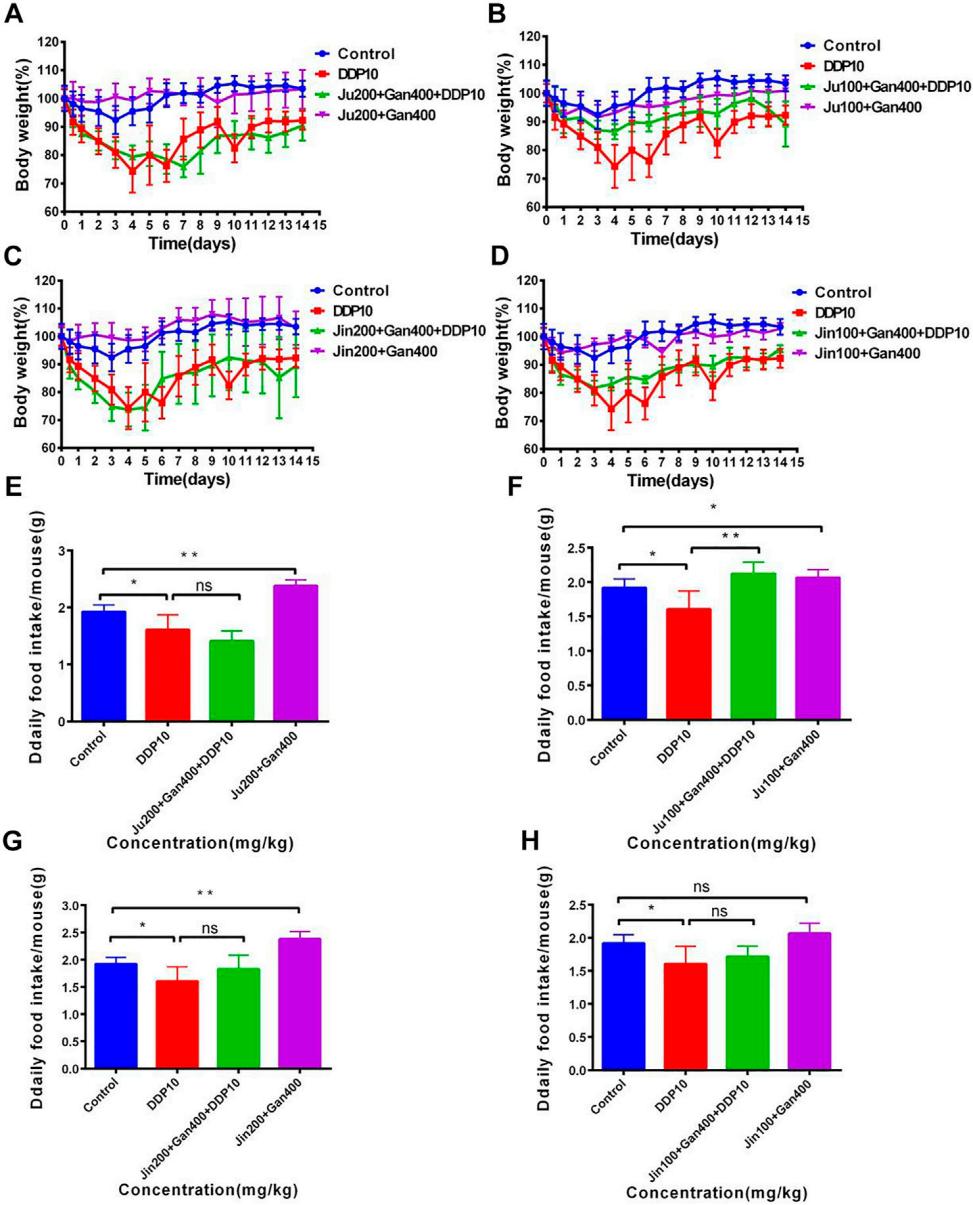
The effect of two Chinese medicine formulas on the decrease of body weight and food intake of mice induced by DDP. **(A–B)** Dose of Ju + Gan on the decrease of body weight of mice induced by DDP. **(C–D)** Dose of Jin + Gan on the decrease of body weight of mice induced by DDP. **(E–F)** Dose of Ju + Gan on the decrease of food intake of mice induced by DDP. **(G–H)** Dose of Jin + Gan on the decrease of food intake of mice induced by DDP. The mean value ±standard error is shown in the figure (*n* = 5). The body weight is represented as mean ± SEM. The *p* values were determined by Student’s t test (*p < .05, **p < .01, ***p < .001).

The weight of the control treatment was maintained at about 100% within 14 days, whereas the weight of mice under DDP treatment represented a continuous decreasing trend and a loss of about 30% was seen on the fifth day. In the next 9 days, the weight of the DDP-treated mice started increasing and was observed as 90% of the original weight at the 14th day posttreatment. Mice receiving individual Chinese herbal formulations (G3-A, G3-B, G3-C, and G3-D) showed similar body-weight trends as compared to the untreated control group, representing the safety of the Chinese herbal formulations. Application of Chinese herbal formulations in + DDP (G4-A, G4-B, G4-C, and G4-D) significantly reversed the sudden weight loss of mice as compared to the DDP group alone (DDP) treatment (Figures 5A–D).

Furthermore, the positive effect of Chinese herbal formulations was also noted on food uptake of mice under the influence of DDP. Findings showed that the food intake of mice was significantly reduced after DDP exposure compared with the nontreated control group (Figures 5E–H) (*p* < .05). The Chinese herbal formulation (G3-A, G3-B, G3-C, and G3-D) treatment significantly increased the food intake of mice compared with the DDP group (*p* < 0.05). Here no significant differences were seen regarding food intake when comparison was made among G4-A, G4-C, G4-D, and DDP treatments (*p* > 0.05). Similarly, the application of G4-B significantly improved the food intake in mice that was reduced by the DDP-alone treatment, whereas the application of G4-A, G4-C, and G4-D showed no significant difference in food intake in mice compared with the DDP-alone treatment (*p* < .01). The abovementioned findings showed that exposure of 100 mg/kg Ju + 400 mg/kg Gan (G4-B) can significantly improve the weight loss and food intake in mice adversely effected by the DDP-alone (DDP) treatment.

### Combined Chinese Herbal Formulations Relieve DDP-Induced Acute Kidney Injury in Mice

**FIGURE 6:**
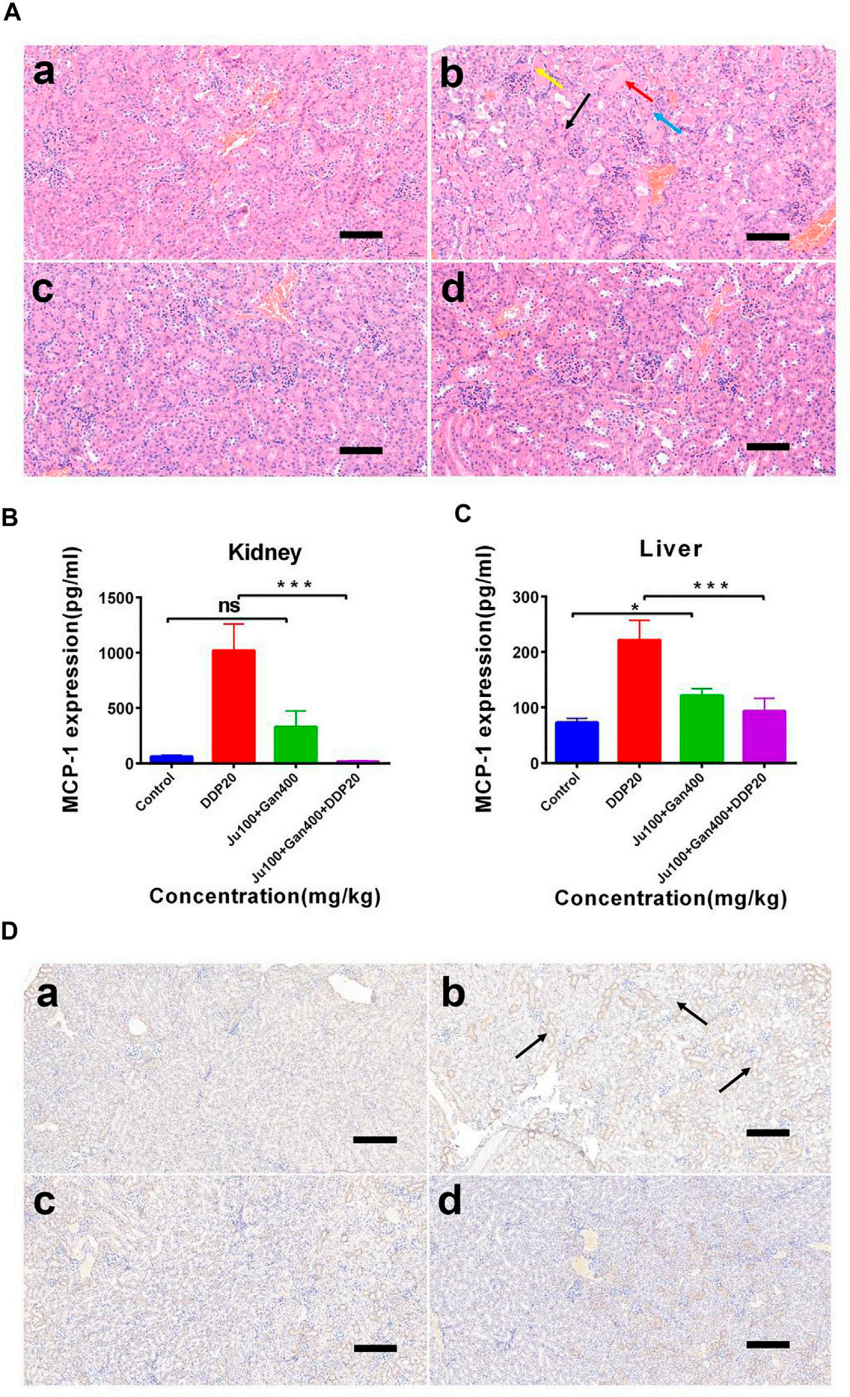
The effect of two Chinese medicine formulas on the nephrotoxicity induced by DDP. **(A)** Representative neutrophil infiltration immunohistochemical staining in mouse kidney: **(A)** control; **(B)** DDP group; **(C)** Chinese medicine formulas group; and **(D)** DDP combined with traditional Chinese medicine formula group. Expression of MCP-1 in kidney **(B)** and in liver **(C)**. **(D)** Representative H&E staining of kidney sections in mice after DDP chemotherapy. **(A)** control; **(B)** DDP group; **(C)** Chinese medicine formulas group; and **(D)** DDP combined with traditional Chinese medicine formula group. Error bars are represented as mean ± SEM. The *p* values were determined by Student’s t test (*p < .05, **p < .01, ***p < .001). Details of experimental procedures are given in *Materials and Methods*.

The H&E analysis showed that the renal medullary cortex of the control group was clearly demarcated, the glomerular capillaries were clearly structured, the structure of renal tubular epithelial cells was normal, and no obvious inflammation was seen (Figure 6A). However, DDP caused cause renal tubular necrosis in the renal cortex (Figure 6A black arrow), tubular expansion (Figure 6A red arrow), necrosis, and shedding of epithelial cell fragments in the lumen (Figure 6A yellow arrow). Here, the renal tubular epithelium was rarely seen under influence of the DDP-alone treatment. Similarly, cellular fatty degeneration and circular vacuoles (Figure 6A blue arrow) were observed in the cytoplasm of epithelial cells after exposure of DDP with no obvious inflammation. Application of G3-B demarcated the renal medullary cortex, whereas the structure of renal tubular epithelial cells was seen normal with no obvious inflammation. G4-B treatment demarcated the renal medullary cortex, whereas the tubular epithelial cell structure was observed as normal with no obvious inflammation. These findings showed that the Chinese herbal formulation consisting of 0.1 g/kg Ju + 0.4 g/kg Gan (G3-B) reduced the damaging effect on the mouse kidney caused by the DDP-alone (DDP) treatment.

We calculated and mapped the MCP-1 expression levels in the liver and kidney of mice (Figures 6B,C). The results show that DDP treatment significantly upregulated the expression of MCP-1 in the liver and kidney (*p* < 0.001). However, the Chinese herbal formulation G3-B (0.1 g/kg Ju + 0.4 g/kg Gan) significantly increased the expression levels of MCP-1 expression in liver tissues (*p* < 0.05), whereas the combined Chinese herbal formulation G4-B (0.1 g/kg Ju + 0.4 g/kg Gan + DDP 10 mg/kg) was able to alleviate the upregulation of MCP-1 expression levels induced by DDP treatment in the liver tissues of mice (*p* < 0.001). There was no significant difference observed in the expression levels of MCP-1 when a comparison was made between the G3-B and control treatments (*p* > 0.05). The combined Chinese herbal formulation G4-B (0.1 g/kg Ju + 0.4 g/kg Gan + DDP 10 mg/kg) can significantly reduce the upregulation of MCP-1 expression levels induced by DDP in the kidney tissues (*p* < .001).

### IHC Analysis

It was also observed that the herbal formulation G3-B reduced kidney damage caused by DDP chemotherapy (Figure 6D). The IHC results showed that DDP treatment promoted the infiltration of neutrophils into the kidney, leading to kidney inflammation and affecting the normal physiological function of the kidney (Figure 6D black arrow). The Chinese herbal formulation G3-B showed no effect on renal neutrophil contents. Nonsignificant differences were denoted in neutrophil content when a comparison was made between combined herbal formulation G4-B and the control group, which proved that the combined Chinese herbal formulation can significantly inhibit the infiltration induced by DDP exposure, thereby protecting the kidney from damage caused by DDP-induced inflammation.

### Blood Cell Analysis

The blood sample was taken on the fourth day posttreatment, centrifuged to obtain serum, and routine blood analysis was performed (Table 6). Findings showed that the number of neutrophils in the control group accounted for 14.86% of the total number of cells. DDP significantly increased the proportion of neutrophils seen as 66.25% (*p* < .001). The traditional Chinese medicine formulation consisting of 0.1 g/kg Ju + 0.4 g/kg Gan (G3-B) had no effect on the proportion of neutrophils in peripheral blood (*p* > .05). The proportion of neutrophils in the combined Chinese herbal formulation (G4-B) was seen as 22.28%, indicating that the G4-B treatment can significantly alleviate the neutrophil levels adversely affected by DDP (DDP) treatment (*p* < .001).

**TABLE 6:** Blood cell analysis.

Detection indicator	Control	DDP20 mg/kg	Ju100 mg/kg + Gan400 mg/kg	Ju100 mg/kg + Gan400 mg/kg + DDP20 mg/kg
Neu (%)	14.86 ± 0.73	66.25 ± 6.95###	13.34 ± 0.80***	22.28 ± 5.90***
Lym (%)	81.98 ± 0.94	30.73 ± 6.56###	84.20 ± 0.70***	74.53 ± 6.49***
Mon (%)	2.03 ± 0.20	1.38 ± 0.21#	1.34 ± 0.14	1.50 ± 0.14
Eos (%)	1.00 ± 0.21	0.75 ± 0.26	0.90 ± 0.29	1.00 ± 0.16
Bas (%)	0.13 ± 0.05	0.90 ± 0.20#	0.22 ± 0.04*	0.70 ± 0.32
WBC (10^9^/L)	5.64 ± 0.72	2.21 ± 0.33##	6.17 ± 0.40***	5.30 ± 0.79**
RBC (10^12^/L)	7.81 ± 0.83	8.19 ± 0.29	7.12 ± 0.39	8.14 ± 0.65
HGB (g/L)	119.50 ± 12.28	126.50 ± 4.56	112.00 ± 5.70	127.00 ± 10.06
HCT (%)	41.48 ± 4.05	42.88 ± 1.59	37.84 ± 2.03	42.58 ± 3.19
MCV (fL)	53.35 ± 0.91	52.38 ± 0.16	52.39 ± 0.40	52.40 ± 0.58

The table shows the mean ± standard error (*n* = 5) by SPSS, software single-factor ANOVA, test (*p < .05 versus DDP, **p < .01 versus DDP, ***p < .001 versus DDP; #*p* < .05 versus control, ##*p* < .01 versus control, ##*p* < .001 versus control).

The number of lymphocytes in the normal saline control group accounted for 81.98% of the total number of cells, and DDP treatment significantly downregulated the proportion of lymphocytes in peripheral blood up to 30.73% compared to the control (*p* < .001). The combined Chinese herbal formulation G4-B did not affect the ratio of lymphocytes in the peripheral blood (*p* > .05). The number of lymphocytes in the G4-B group accounted for 74.53% of the total number of cells. The combined Chinese herbal formulation G4-B (0.1 g/kg Ju + 0.4 g/kg Gan + DDP 10 mg/kg) significantly improved the proportion of lymphocytes that were seen as adversely affected by DDP treatment in mice (*p* < .001). The number of white blood cells in the peripheral blood was as 5.64 × 10^9^/l in the control group. Here DDP significantly decreased the number of white blood cells up to 2.21 × 10^9^/l. The Chinese herbal formulation consisting of 0.1 g/kg Ju + 0.4 g/kg Gan (G3-B) showed no significant change in the number of white blood cells of the peripheral blood system. The combined Chinese herbal formulation G4-B significantly increased the white blood cell count up to 2.4-fold compared to the DDP-alone treatment (*p* < .01).

Alongside, the combined Chinese herbal formulation G4-B consisting of 0.1 g/kg Ju + 0.4 g/kg Gan + DDP 10 mg/kg alleviated a number of abnormalities in blood cells caused by the DDP-alone (DDP) treatment, proving the positive role of the Chinese herbal formulation during chemotherapy.

### Blood Biochemical Analysis

We used the serum for blood biochemical tests. The results presented in Table 7 showed that the combined Chinese herbal formulation G4-B (0.1 g/kg Ju + 0.4 g/kg Gan + DDP 10 mg/kg) significantly downregulates the ALT value compared with the DDP-alone and control groups (*p* < .05). It was also seen that the DDP-alone (221 U/l) treatment significantly increased the AST value as compared to control (162.0 U/l) (*p* < 0.01) The Chinese herbal formulation G3-B and the combined formulation G4-B can significantly downregulate the AST value up to (107.40 and 94.50 U/l), respectively, as compared to the DDP-alone treatment (221 U/l) (*p* < 0.01). DDP treatment significantly upregulated the UREA activity up to 8.7-fold as compared to the control (*p* < 0.001). Here Chinese herbal formulation G3-B showed a nonsignificant effect on UREA activity as compared to control, whereas the combined Chinese herbal formulation G4-B significantly decreased UREA activity up to 9.3-fold compared with the DDP-alone treatment (*p* < 0.001).

**TABLE 7:** Blood biochemical analysis.

Detection indicator	Control	DDP20 mg/kg	Ju100 mg/kg + Gan400 mg/kg	Ju100 mg/kg + Gan400 mg/kg + DDP20 mg/kg
ALT (U/L)	41.25 ± 6.42	44.75 ± 3.84	29.00 ± 3.21*	30.25 ± 1.75*
AST (U/L)	162.0 ± 19.78	221.0 ± 55.30##	107.40 ± 9.84**	94.50 ± 9.26**
ALP	81.75 ± 10.83	79.75 ± 18.96	69.40 ± 14.07	72.25 ± 10.77
UA	41.00 ± 13.77	15.00 ± 2.20#	20.26 ± 1.14	18.13 ± 0.58
UREA (mmol/L)	7.33 ± 0.72	62.88 ± 2.96###	6.80 ± 0.59***	6.83 ± 0.41***
CREA (μmol/L)	3.33 ± 0.33	89.0 ± 22.11###	3.12 ± 1.15***	3.05 ± 0.64***
DBIL	0.28 ± 0.13	0.13 ± 0.06	0.22 ± 0.06	0.23 ± 0.13
TP	38.18 ± 2.05	34.35 ± 1.63	38.46 ± 1.76	37.00 ± 1.54
ALB	23.50 ± 1.26	18.38 ± 1.18#	21.88 ± 1.38	22.15 ± 0.74
GLO	15.18 ± 0.84	15.98 ± 0.63	16.58 ± 1.17	14.85 ± 0.85

The table shows the mean ± standard error (*n* = 5) by SPSS, software single-factor ANOVA, test (*p < .05 versus DDP, **p < .01 versus DDP, ***p < .001 versus DDP; #*p* < .05 versus control, ##*p* < .01 versus control, ##*p* < .001 versus control).

DDP treatment increased the CREA value up to 26-fold as compared to the control (*p* < .001). The Chinese herbal formulation G3-B (0.1 g/kg Ju + 0.4 g/kg Gan) had no significant effect on CREA value compared with the control (*p* > .05). However, the combined Chinese herbal formulation G4-B significantly decreased the CARE value up to 25-fold compared with the DDP-alone treatment (*p* < .001).

### Effects of Different Nature Compounds From Ju and Gan on the Viability of Lung Cancer Cells

**FIGURE 7:**
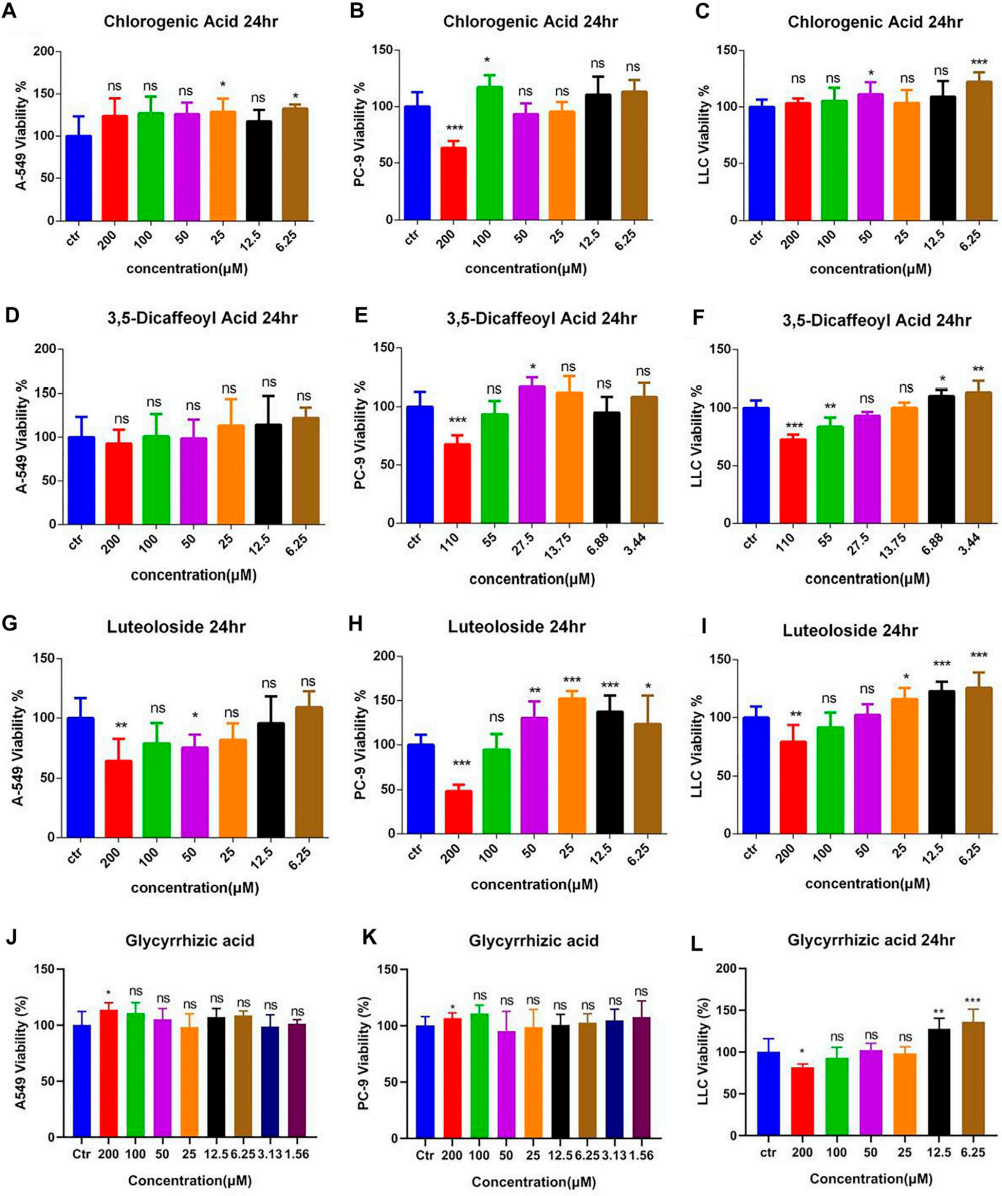
The effects of different compound on the viability of lung cancer cells. **(A–C)** Dosage of chlorogenic acid on the antitumor activity of DDP in A549, PC-9, and LLC cells. **(D–F)** Dose of 3,5-dicaffeoylquinic acid on the antitumor activity of DDP in A549, PC-9, and LLC cells. **(G–I)** Dosage of luteoloside on the antitumor activity of DDP in A549, PC-9, and LLC cells. **(J–L)** Dosage of glycyrrhizic acid on the antitumor activity of DDP in A549, PC-9, and LLC cells. Error bars are represented as mean ± SEM. The *p* values were determined by Student’s t test (*p < 0.05, **p < 0.01, ***p < 0.001 versus control).

The antitumor effect of different purified compounds 3-CQA, 3,5-diCQA, LUS, and glycyrrhizic acid (GA) present in Ju and Gan was seen in combination with DDP. The results (Figures 7A–C) of the viability test showed that 3-CQA did not inhibit the viability of the three lung cancer cell lines (A549, PC-9, LLC cells) at a concentration of 100 μM. However, 3-CQA significantly inhibited the viability of PC-9 cells at a concentration of 200 μM (Figure 7B) (*p* < .001). In case of A549 and LLC cells, a lower concentration of 3-CQA (6.25 μM) showed an increase in the cell viability, up to 132.63% and 122.29% as compared to the control group, respectively (*p* < .05). Similarly, 3,5-diCQA (Figures 7E,F) showed a significant inhibitory effect on the cell viability of LLC cells and PC-9 cells at a concentration of 110 μM compared to the control group (*p* < .001), whereas a nonsignificant effect was seen on the viability of A549 cells (Figure 7D) (*p* > 0.05 versus control). A low concentration of 3,5-diCQA (3.44 μM) can increase the viability of LLC cells up to 133.45% compared with the control group (Figure 7F) (*p* < 0.01). LUS has a significant inhibitory effect on the viability of three different (A549, LLC, PC-9) lung cancer cell lines at up to 36.23%, 31.97%, and 51.59% respectively, when applied at a concentration of 200 μM (Figures 7G–I) (*p* < 0. 01), whereas a non-significant effect on the viability of cancer cell lines was seen when LUS was applied at a concentration of 100 μM (*p* > 0.05). At lower concentrations of LUS (25 μM, 12.5 μM, 6.25 μM), the cell viability of both LLC and PC-9 cells was improved significantly compared to the control group (*p* < .05). The highest increase of 125.70% was seen in cell viability when LUS was applied at 6.25 μM as compared to the control group (*p* < . 001), whereas, in the case of PC-9 cells, the highest increase of 152.38% was seen when LUS was applied at 25 μM as compared to the control group (*p* < . 001).

For LLC cells, 200 μM GA (Figure 7L) significantly inhibited cell viability up to 18.17% as compared to the control group (*p* < 0. 05), whereas, when applied at lower concentrations of 12.5 and 6.25 μM, GA significantly increased the viability of LLC cells up to 127.42% and 135.98%, respectively, as compared to the control group (*p* < . 0001). Regarding A549 and PC-9 cells (Figures 7J,K), 200 μM GA significantly increased cell viability up to 113.54% and 106.56%, respectively, as compared to the control group (*p* < . 05). The other concentrations of GA had no effect on cell viability.

### The Effect of Natural Compounds From Ju or Gan Alone Combined With DDP on the Viability of Lung Cancer Cells

Preliminary findings show that the high concentration of the freeze-dried powder of Ju increased the inhibitory effect of DDP on tumor cells (Figure 2). Based on the findings of the previous experiment, we selected 3-CQA, 3,5-diCQA, and LUS combined with DDP to further assess their antitumor capabilities.

**FIGURE 8:**
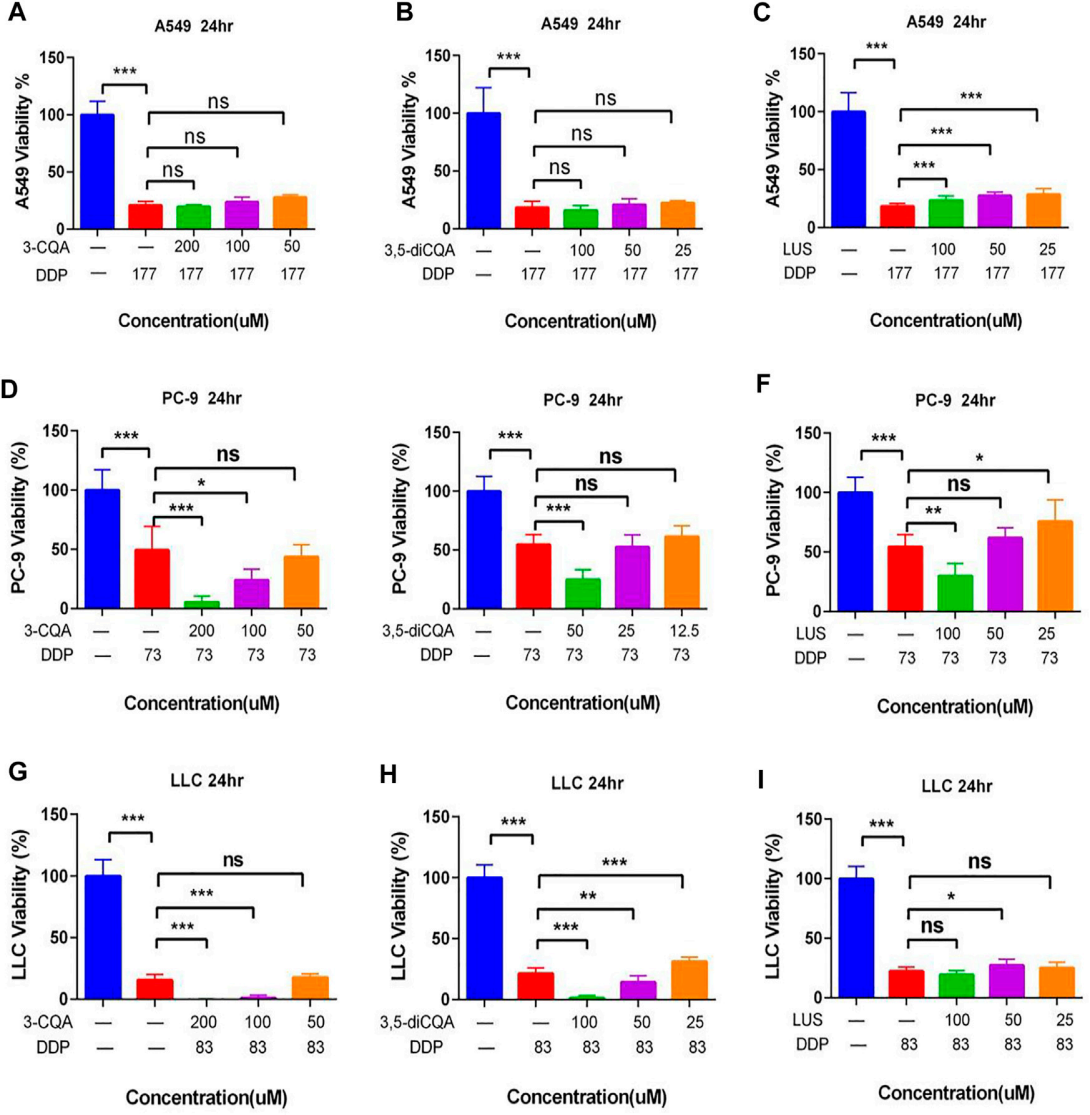
Effects of different compound on the antitumor activity of DDP *in vitro*. **(A–C)** Dosage of chlorogenic acid (3-CQA), 3,5-dicaffeoylquinic acid (3,5-diCQA), or luteoloside (LUS) on the antitumor activity of DDP in A549 cells. **(D–F)** Dosage of chlorogenic acid (3-CQA), 3,5-dicaffeoylquinic acid (3,5-diCQA), or luteoloside (LUS) on the antitumor activity of DDP in PC9 cells. **(G–I)** Dosage of chlorogenic acid (3-CQA), 3,5-dicaffeoylquinic acid (3,5-diCQA), or luteoloside (LUS) on the antitumor activity of DDP in LLC cells. Error bars are represented as mean ± SEM. The *p* values were determined by Student’s t test (*p < 0.05, **p < .01, ***p < .001).

Based on the antitumor potential of single compounds against A549 cell proliferation, the combined initial concentrations of 3,5-diCQA, 3-CQA, and LUS were adjusted to 200 and 100 μM, respectively. Results (Figures 8A–C) showed that the three compounds (3,5-diCQA, 3-CQA, and LUS) found in Ju when applied combined with DDP failed to enhance the inhibitory effect of DDP against A549 cell viability. Among them, LUS even reduces the efficacy of DDP (Figure 8C). For PC-9 cells, 3-CQA (100–200 μM) increased the inhibitory effect of DDP on cell viability up to 25.1% and 43.9% as compared to DDP alone (Figure 8D) (100 μM, *p* < .001; 200 μM, *p* < 0.05). 50 μM of 3,5-diCQA significantly increased the inhibitory effect of DDP on cell viability up to 29.7% as compared to DDP alone (Figure 8E) (*p* < 0.0001). Similarly, 100 μM of LUS increased the inhibition rate of DDP up to 15.4% as compared to DDP alone (*p* < 0.001) while 25 μM of LUS reduced the antitumor properties of DDP to PC-9 cells (Figure 8F) (*p* < 0.05). For LLC cells, 3-CQA (100–200 μM) and 3,5-diCQA (50–100 μM) obviously increased the inhibitory effect of DDP against LLC cells (Figures 8G,H) (*p* < 0.01). However, 25 μM of 3,5-diCQA conversely decreased the inhibitory effect of DDP on LLC cells (Figure 8H) (*p* < 0.01 versus DDP). In addition, the antitumor effect of LUS on LLC cells was observed in A549 cells at the concentration of 50 μM exclusively (Figure 8I) (*p* < 0. 05).

As LUS adversely affected the antitumor properties of DDP, it was omitted from further downstream studies. 3-CQA, 3,5-diCQA, and GA increased the antitumor properties of DDP, so these two compounds were selected for downstream analysis.

### The Effect of Two Natural Compounds Combined With DDP on the Viability of Lung Cancer Cells

**FIGURE 9:**
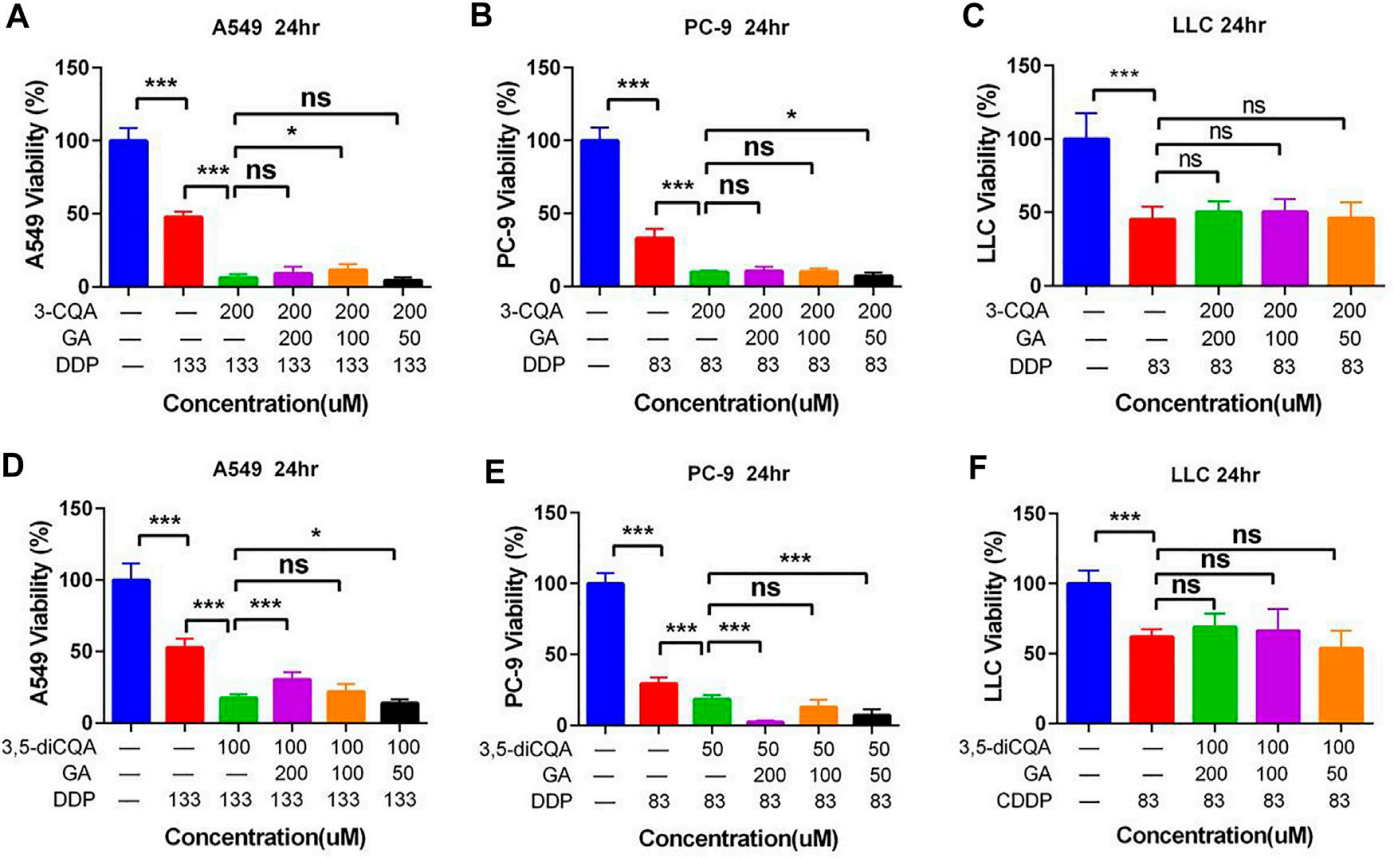
The effect of two natural compounds combined with cisplatin on the viability of lung cancer cells. **(A–C)** Chlorogenic acid (3-CQA) combined with glycyrrhizic acid (GA) on the antitumor activity of DDP in A549, PC9, and LLC cells. **(D–F)** 3,5-Dicaffeoylquinic acid (3,5-diCQA) combined with glycyrrhizic acid (GA) on the antitumor activity of DDP in A549, PC9, and LLC cells. Error bars are represented as mean ± SEM. The *p* values were determined by Student’s t test (*p < 0.05, **p < 0.01, ***p < 0.001).

Preliminary studies showed that Gan combined with Ju increased the antitumor properties of DDP (Figure 2). Hence, two active constituents of Ju (3-CQA) and Gan (glycyrrhizin acid) were used in combination with DDP to examine antitumor properties. The results showed that 3-CQA and GA effectively increased the antitumor properties of DDP against A549 cells and PC-9 cells (Figures 9A,B). In addition, GA enhanced the anti-PC-9 effect of 3-CQA combined with DDP only at 50 Μm (*p* < .05 versus 3-CQA + DDP) (Figure 9B), whereas addition of 3-CQA and GA was of no use to increase the antitumor properties of DDP against LLC cells (Figure 9D) (*p* > 0.05 versus DDP).

The addition of 3-CQA and GA increased the antitumor properties of DDP in the case of both A549 cells and PC-9 cells (Figures 9A,B,D,E). Similarly, the antitumor properties of DDP were significantly increased against A549 cells and PC-9 cells, when used in combination with 3,5-diCQA and GA (Figures 9D,E). In the case of PC-9 cells, the combined use of 3,5-diCQA (50 μM) and GA (50 and 200 μM) further increased the antitumor properties of DDP (Figure 9E) (*p* < . 001 versus DDP). The combined use of 3-CQA or 3,5-diCQA and GA along with DDP showed no increase in antiproliferating properties of DDP against LLC cells (Figures 9C,F) (*p* > . 05 versus DDP).

### Inhibitory effects of two natural compounds in combination with DDP on tumors *in vivo*

LLC cells were inoculated under the left axillary skin of C57BL/6 mice to establish a mouse lung cancer tumor model. The tumor mass volume of mice was measured by percentage, and the average tumor volume of mice on the first day was considered as 100% in each group (Day 0). Furthermore, the change of the average tumor volume of each group was monitored up to 14 days.

**FIGURE 10:**
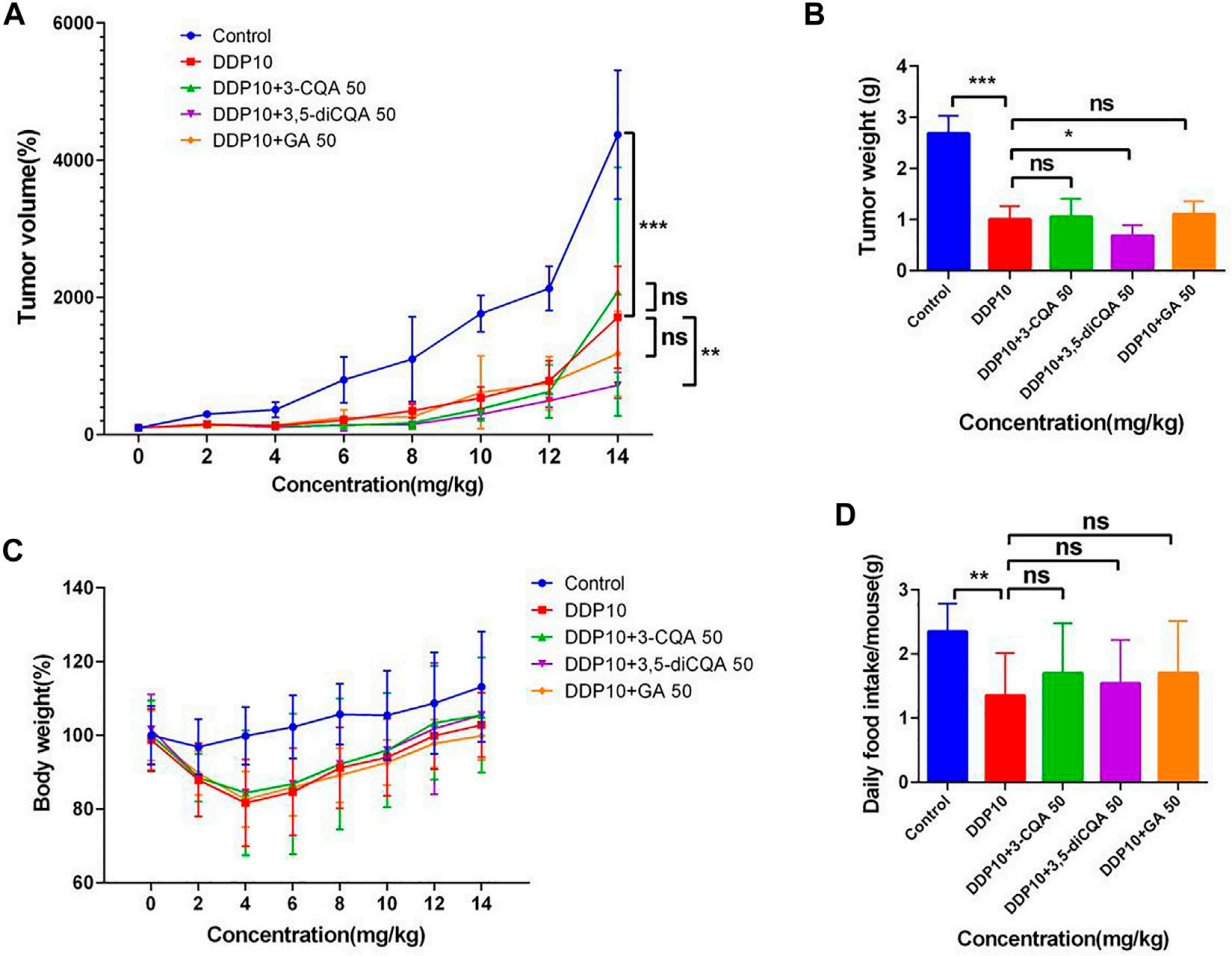
Effects of different compound on the antitumor activity of DDP *in vivo*. **(A)** Tumor growth trends in different groups of mice. **(B)** Different groups of mouse tumor weight in the end of the experiment. **(C,D)** Changes in body weight and food intake of mice in different groups. Error bars are represented as mean ± SEM. The *p* values were determined by Student’s t test (*p < .05, **p < .01, ***p < .001). Details of experimental procedures are given in *Materials and Methods*.

The tumor growth rate of DDP-treated groups was significantly reduced compared with control (Figures 10A,B) (*p* < .001). On the 14th day, the tumor volume in the control group had increased 43-fold compared to the initial value, and the average weight of tumor mass reached up to 2.68 g. The tumors in the DDP group increased up to 17-fold compared to the initial value, with an average weight gain of 1.01 g. The volume of the tumor under treatment with 3-CQA, 3,5-diCQA, and GA increased up to 20-, 7-, and 12-fold, respectively, compared to the initial value, respectively. Similarly, the average tumor weight was increased up to 1.05, 0.69, and 1.11 g, respectively, in the same regard. The application of 3,5-diCQA + DDP significantly reduced the tumor weight (*p* < .01) and volume (*p* < .05) as compared to the DDP-alone treatment. No significant difference was observed when 3-CQA and GA were used in combination with DDP as compared to the DDP-alone treatment (*p* > .05).

Along with the tumor weight and volume, a change in the body weight and food intake of mice was also monitored in different treatment groups. Considering the initial weight of each treatment as 100%, the subsequent weight changes relative to the initial value were monitored. The results showed (Figure 10C) that the weight of the mice continued to decrease after chemotherapy. On the fifth day, the weight of the mice reached the lowest value and then started increasing in subsequent days. At the end of the experiment, the lowest body weight (99.8%) was observed under treatment of DDP + GA as compared to the rest of the treatments. This was followed by the DDP-alone treatment which represented 102.76% body weight compared to the initial value, whereas both treatments DDP + 3-CQA and DDP + 3,5-diCQA showed a 105% body weight compared to the initial value. The weight of the control group increased up to 113.13% of the initial value. As the control group did not receive chemotherapy drugs, the tumor volume increased very rapidly, and the proportion of tumor weight in the body weight of mice was higher (average tumor weight 2.68 g) as compared to the rest of the treatments.

The result of the daily food intake of mice under each treatment showed that the food intake of mice was significantly reduced after exposure of DDP (Figure 10D) (*p* < .01). The food intake was reduced from 2 g to less than 1 g and started increasing in subsequent days. By the end of the experiment, the food intake of the mice receiving active constituents of herbal formulations along with DDP was higher than that of the control treatment. The average food intake of the mice receiving active constituents of herbal formulation along with DDP was relatively higher with nonsignificant differences as compared to DDP alone (*p* > .05). The average daily food intake of mice in the control group was 2.37 g, whereas under DDP treatment the average daily food intake of mice was 1.97 g. The food intake of mice under the rest of the treatments was higher than that with DDP alone.

### Protective Effects of Different Concentrations of Compounds From Ju or Gan on Acute Kidney Injury Induced by DDP

The results of previous studies have shown that the aqueous extracts of Ju (100 mg/kg) and Gan (400 mg/kg) can effectively alleviate weight loss, appetite loss, and kidney damage induced by DDP in mice (Figures 5, 6). In the early stage of this study, the contents of 3-CQA and 3,5-diCQA in freeze-dried powdered material of Ju and GA in freeze-dried powder of Gan were determined.

**FIGURE 11:**
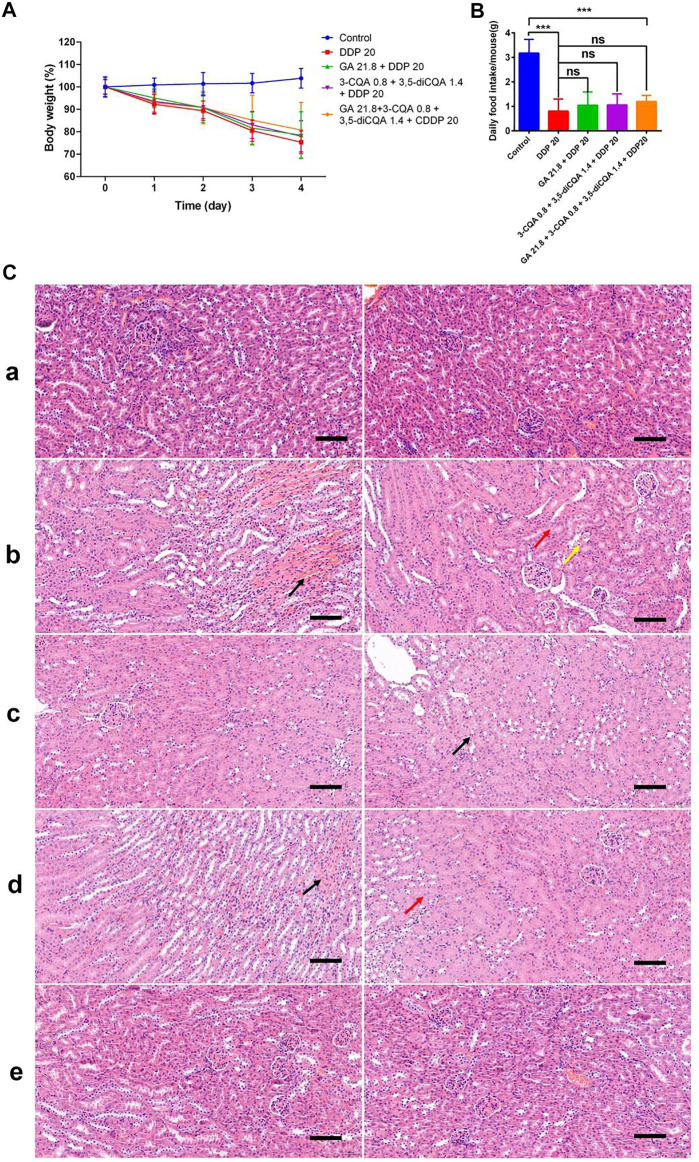
The effect of two Chinese medicine formulas on the nephrotoxicity induced by DDP. **(A)** Body weight of mice in different groups. **(B)** Food intake of mice in different groups. The mean value ± standard error is shown in the figure (*n* = 5). The body weight is represented as mean ± SEM. The *p* values were determined by Student’s t test (*p < 0.05, **p < 0.01, ***p < 0.001). **(C)** Representative H&E staining of kidney sections in mice after DDP chemotherapy. **(A)** control; **(B)** DDP group; **(C)** DDP + GA group; **(D)** DDP+3-CQA+3,5-diCQA group; and **(E)** DDP+3-CQA+3,5-diCQA + GA group.

The initial body weight of all groups was set to 100%, and the changes in the body weight of the mice in each group relative to the initial value were monitored. Body weight analysis (Figure 11A) showed that the weight of the mice continued to decrease after the injection of DDP. Four days after the injection of DDP, the body weight was decreased up to 75.32%, 78.0%, and 78.51% under treatment with DDP alone (DDP 20), the two-drug group (GA 21.8 + DDP 20), and the three-drug group (3-CQA 0.8 + 3,5-diCQA 1.4 + DDP 20), respectively, whereas the four-drug group (GA 21.8 + 3-CQA 0.8 + 3,5-diCQA 1.4 + DDP 20) displayed the best weight amelioration, with 80.74% of the initial body weight.

The average daily food intake of the control group reached up to 3 g, whereas the daily food intake of the mice under DDP treatment was less than 1.5 g (Figure 11B). Except for the control group, the mice receiving the four-drug group treatment had the highest average food intake. Especially on the third day, the average food intake per mouse in the four-drug group was 1.06 g, which was much higher as compared to 0.32 g in the DDP-alone treatment. On the fourth day, the average food intake of the DDP-alone treatment was 0.44 g, whereas under the two-drug, three-drug, and four-drug treatments, the average food intake was 0.83, 0.89, and 0.93 g, respectively. These food intake data were consistent with the change in body weight.

### Blood Cell Analysis

Blood analysis (Table 8) showed that DDP treatment significantly increased the percentage of neutrophils (Neu%) (*p* < .01), monocytes (Mon%) (*p* < .05), and eosinophils (Eos%) (*p* < .01) in peripheral blood of mice but significantly downregulated the percentage of lymphocytes (Lym%) (*p* < .01), number of white blood cells (WBC) (*p* < .001), neutrophils (Neu) (*p* < .05), and lymphocytes (Lym) (*p* < .01) compared to the control group. The combined use of the compounds (GA, 3-CQA, 3,5-diCQA, DDP 20) along with DDP showed significant ameliorative effects on abnormal indicators such as the proportion of monocytes (*p* < .05), the proportion of eosinophils (*p* < .05), the number of white blood cells (*p* < .01), and neutrophils (*p* < .05) compared to the DDP group.

**TABLE 8:** Blood cell analysis.

Detection indicator	Control	DDP 20 (mg/kg)	GA 21.8 (mg/kg)	3-CQA 0.8 + 3,5-diCQA 1.4 (mg/kg)	GA 21.8 + 3-CQA 0.8 + 3,5-diCQA 1.4 (mg/kg)
NEU (%)	17.23 ± 1.03	28.1 ± 1.35##	33.53 ± 4.53##	38.93 ± 5.33##	27.2 ± 6.16
Lym (%)	79.87 ± 1.47	67.6 ± 1.27##	64.33 ± 3.72	57.47 ± 5.59###	67.5 ± 4.42
Mon (%)	1.6 ± 0.12	3.23 ± 0.48#	1.07 ± 0.23*	1.57 ± 0.09*	3.3 ± 0.36#
Eos (%)	0.9 ± 0.12	3.87 ± 0.68#	0.9 ± 0.15*	1.83 ± 0.18*	2.73 ± 0.49#
Bas (%)	0.3 ± 0.06	0.37 ± 0.07	0.37 ± 0.03	0.2 ± 0	0.47 ± 0.03
WBC (10^9^/L)	4.25 ± 0.07	1.43 ± 0.22###	2.02 ± 0.08##	1.86 ± 0.23###	2.78 ± 0.27##*
RBC (10^12^/L)	8.41 ± 0.14	7.57 ± 0.27	7.13 ± 0.82	7.63 ± 0.32	7.48 ± 0.29
HGB (g/L)	126 ± 0.58	118 ± 4.04	109.33 ± 14.71	119.67 ± 4.67	116 ± 3.06
HCT (%)	42.17 ± 0.75	36.93 ± 1.16	35.2 ± 3.7	36.47 ± 1.59	35.13 ± 1.06
MCV (fL)	50.13 ± 0.09	49.1 ± 0.12	49.73 ± 0.33	48.6 ± 0.46	49.53 ± 0.15
NEU (10^9^/L)	0.79 ± 0.1	0.4 ± 0.05#	0.73 ± 0.04**	0.67 ± 0.16**	0.54 ± 0.01#
Lym (10^9^/L)	3.47 ± 0.11	1.33 ± 0.23##	1.34 ± 0.1##	0.99 ± 0.15###	1.69 ± 0.12##
Mon (10^9^/L)	0.06 ± 0.01	0.03 ± 0.01	0.02 ± 0.01	0.03 ± 0.01	0.09 ± 0.01*
Eos (10^9^/L)	0.04 ± 0.01	0.06 ± 0.01	0.01 ± 0*	0.03 ± 0	0.07 ± 0.01
Bas (10^9^/L)	0.01 ± 0	0 ± 0	0 ± 0	0 ± 0	0.01 ± 0

The table shows the mean ± standard error (*n* = 5) by SPSS, software single-factor ANOVA, test (*p < .05 versus DDP, **p < .01 versus DDP, ***p < .001 versus DDP; #*p* < .05 versus control, ##*p* < 0.01 versus control, ##*p* < .001 versus control).

### Blood Biochemical Analysis

Blood biochemical analysis (Table 9) showed that DDP treatment significantly increased AST (*p* < .01 versus control) and CREA (*p* < .001 versus control), whereas a nonsignificant effect was seen for UREA (*p* > .05 versus control). Among them, creatinine and urea are important indicators for evaluating kidney function. The exposure of DDP along with different compounds did not significantly inhibit the increase of the abovementioned blood parameters. When the aqueous extracts of chrysanthemum and licorice were directly applied to this model in the early stage, both of them significantly inhibited the upregulation of DDP-induced AST (*p* < .01 versus DDP), CREA (*p* < .001 versus DDP), UREA (*p* < .001 versus DDP), and other biochemical indicators (Table 7).

**TABLE 9:** Blood biochemical analysis.

Detection indicator	Control	DDP 20 (mg/kg)	GA 21.8 (mg/kg)	3-CQA 0.8 + 3,5-diCQA 1.4 (mg/kg)	GA 21.8 + 3-CQA 0.8 + 3,5-diCQA 1.4 (mg/kg)
ALT (U/L)	45.03 ± 1.5	36.73 ± 4.85	37.4 ± 3.32	35.98 ± 2.73	44.77 ± 7.3
AST (U/L)	122.8 ± 9.92	179.87 ± 8.47##	152.45 ± 12.27	148.68 ± 9.96	174.57 ± 32.02
CREA (μmol/L)	0.1 ± 0.29	5.63 ± 0.37###	4.95 ± 2.8	2.73 ± 1.43	3.47 ± 1.55
UREA (mmol/L)	8.77 ± 0.13	11.78 ± 1.52	17.35 ± 4.98	13.11 ± 2.16	13.75 ± 3.29

The table shows the mean ± standard error (*n* = 5) by SPSS, software single-factor ANOVA, test (*p < .05 versus DDP, **p < .01 versus DDP, ***p < .001 versus DDP; #*p* < .05 versus control, ##*p* < .01 versus control, ##*p* < .001 versus control).

In addition, the H&E staining results (Figure 11C) of tissue sections showed that the control group and the four-drug group had clear demarcations in the cortex and medulla, abundant renal tubules, tightly arranged, normal epithelial cells, and relatively small glomerular capillaries. No obvious abnormalities were seen regarding the mentioned anatomical parameters. In the DDP-alone treatment, the cortex and medulla of the tissue were clearly demarcated, and the renal tubules were abundant and tightly arranged. Interstitial congestion was seen at the junction of the cortex and medulla (Figures 11B,C, black arrow). More renal tubular epithelial cells were swollen, and the cytoplasm was weak (Figures 11B,C, red arrow). A small amount of renal tubular epithelial cell necrosis, nuclear fragmentation (Figures 11B,C, yellow arrow), and glomerular capillaries were clearly visible, whereas no other obvious abnormalities were seen. In the double-drug group (GA 21.8 + DDP 20), the renal tubules were abundant in tissues and arranged tightly. A small amount of renal tubular epithelial cells was swollen (Figure 11C, black arrow). The glomerular capillaries were clear with no other obvious abnormalities. In the three-drug group (3-CQA 0.8 + 3,5-diCQA 1.4 + DDP 20), the cortex tissue and medulla were clearly demarcated, and renal tubules were found abundantly and tightly arranged. Interstitial congestion was seen at the cortex and medulla junction (Figures 11C,D, black arrow). A small amount of renal tubular epithelial cells was swollen (Figures 11C,D, red arrow), the cytoplasm was loose and lightly stained, and the glomerular capillaries were clear with no obvious abnormalities.

In general, the combination of compounds or dried aqueous extract of Ju and Gan can improve the weight and appetite loss and blood cell abnormalities caused by DDP chemotherapy in mice. However, the purified compounds were found far less effective as compared to the aqueous extracts.

## Discussion

Platinum drugs have been the most extensively used for cancer treatment in the past 50 years (Sun et al., 2019). DDP is widely used to cure a series of cancers including lung, prostate, ovarian, brain, and breast cancer (Dasari and Tchounwou, 2014). DDP-based chemotherapy is commonly used in the treatment of lung cancer of the second and third stages (Masutani et al., 1996; Pignon et al., 2008; Kim et al., 2019). However, the use of platinum-based drugs has been limited due to adverse reactions such as nausea, vomiting, anorexia, and weight loss (Francescato et al., 2004). Nephrotoxicity is considered as the main dose-limiting side effect of DDP (Sastry and Kellie, 2005; Changizi-Ashtiyani et al., 2016). Nearly 20%–30% of cancer patients received DDP chemotherapy accompanied by severe acute kidney injury (Frezza et al., 2010). Therefore, it is of immense importance to find novel approaches to reduce the toxic effects of chemotherapy drugs.

In immunology, inflammation is the defensive response of the body after recognizing infection and injury (Ashley et al., 2012). More attention has been paid to inflammation in the presence of nephrotoxicity caused by DDP. An increase in the expression of tumor necrosis factor-a (TNF-α, a typical inflammatory cytokine) has been confirmed in a mouse model of DDP-induced nephrotoxicity (Kelly et al., 1999). In addition, TNF-α inhibitors can reduce DDP-induced kidney damage and renal dysfunction in mice. Compared to wild-type mice, TNF-α knockout mice showed reduced kidney damage and higher survival rates (Ramesh and Reeves, 2002). Previous studies have proved that acute kidney injury induced by TNF-α in DDP is closely related to a variety of cytokines in the kidney. The study performed by Deng et al. (2001) showed that the exogenous IL-10 can inhibit the upregulation of TNF-α and intercellular adhesion factor ICAM-1 caused by DDP and prevent the influx of neutrophils into the kidney induced by DDP (Deng et al., 2001).

Immune cells also undergo significant changes after DDP administration. Tadagavadi et al. (2010) showed the DDP-induced neutrophil infiltration inside the kidney (Tadagavadi and Reeves, 2010a). However, Faubel et al. (2007) proposed that DDP-induced renal insufficiency can be reduced by effective removal of renal neutrophils (Faubel et al., 2007). In other words, neutrophils can only reflect the degree of kidney damage induced by DDP, rather than the underlying cause. The role of macrophages in DDP-induced nephrotoxicity remains unclear. Studies have shown that the number of macrophages in the kidney increases by a factor of two after DDP administration (Lu et al., 2008). Regulatory T cells can regulate the functions of other immune cells. When regulatory T cells were transferred to T cell-deficient mice and wild-type mice, the renal insufficiency and mortality induced by DDP were significantly reduced (Lee et al., 2008; Cao et al., 2010). This indicates that regulatory T cells play a protective role against DDP-induced kidney injury. Dendritic cells are the sentinels of the immune system and play an important role in the immune system. Studies have shown that dendritic cells reduce DDP-induced nephrotoxicity and related inflammation (Tadagavadi and Reeves, 2010b).

In 2010, Zheng et al. found that a four-herb Chinese medicine formulation (PHY906), first described 1,800 years ago, effectively decreased gastrointestinal toxicity induced by the chemotherapeutic drug CPT-11 (Lam et al., 2010). In a previous research, we have proved the anti-inflammatory ability of Ju, Jin, and Gan using the cell inflammation model (Wang et al., 2021). Based on the findings of our previous study, we analyzed the effect of different doses of the three aforementioned herbs in tumor treatment. Cell studies showed that Jin (0.4 mg/ml) significantly enhanced the inhibitory effect of DDP on the proliferation of PC-9, H1299, LLC, and A549 cells (Figure 2). Similarly, Ju (0.4 mg/ml) also significantly increased the inhibitory effect of DDP against PC9, LLC, and A549 lung cancer cells (Figure 2). Our previous study has shown that a single treatment with Ju or Jin significantly reduced the mouse body weight, and a combination of different traditional Chinese medicine (TCM) helped with maintaining the body weight (Wang et al., 2021), which correlates with the common logistics of TCM formulation. Abiding by the same philosophy, we tested groups of the TCM formula for the antitumor activity in the presence of DDP. Ju and Jin at a concentration of 0.4 mg/ml or lower showed no synergistic inhibitory effect in combination with DDP. Gan was unable to improve the anti-proliferating effect of DDP against tumor cells under both high and low concentrations (Figure 3). After administrating mice with Ju and Gan or Jin decoction of traditional Chinese medicine, we found that these herbal formulations did not significantly promote the antitumor effects of DDP *in vivo* (Figure 4), indicating that the herbal formula was not able to synergistically with DDP in killing the tumor. The result did not correlate with a previous study, in which a TCM formula enhanced the therapy of a compound CPT-11, although the formula also mainly targeted the inflammation brought by the chemical drug (Lam et al., 2010). Further investigation may be needed to look into the discrepancy.

We then asked if our herbal formula might have contributed to the nephrotoxicity, which is common during DDP treatment. A mouse model of acute kidney injury induced by DDP was established and found that the formulation based on Ju (100 mg/kg) and Gan (400 mg/kg) significantly alleviated the antitumor properties of DDP in tumor mouse models along with reduced side effects. The sudden weight loss and reduction in food intake caused by DDP treatment (Alhadeff et al., 2017) were also improved by combination with the formula (Figure 5). H&E tissue section staining and immunohistochemical analysis showed that combined formulation based on DDP and herbal formulation (G4-B) effectively reduced DDP-induced renal tissue damage and renal inflammatory cell infiltration along with the reduced expression of MCP-1 in liver and kidney tissues induced by DDP (Figure 6).

In the next phase of the study, the main metabolic components of Ju and Gan were examined for their role to reduce acute kidney injury induced by DDP. For that purpose, 3-CQA, 3,5-diCQA, LUS, and GA were combined with DDP and used for antitumor studies both *in vitro* and *in vivo*. The results showed that 3-CQA and 3,5-diCQA enhanced the inhibitory effect of DDP on PC-9 and LLC cells, whereas LUS reduced the antitumor effect of DDP. GA was unable to further increase the synergistic antitumor effect of 3-CQA and 3,5-diCQA against tumor cells (Figures 7, 8). The combination of 3,5-diCQA and Gan assisted the cytotoxicity in PC-9 cancer cells and also in mouse treatment (Figures 9, 10), but the rest of the combination neither showed significance, nor did the same combination to A549 and LLC cells, indicating that the major components of the herbal medicine, consistently acting as the herbs themselves, do not have strong antitumor activity. We also observed that 3-CQA, 3,5-diCQA, and GA did not alleviate the weight, appetite, and abnormal blood cells in a mouse model of acute kidney injury induced by DDP, although they are far less effective rather than the combined decoction of traditional Chinese formulations. In all, on top of our previous work of an anti-inflammatory herbal medicine quantitative screening platform, we concluded that a few kinds of anti-inflammatory TCM are capable of reducing the DDP-induced nephrotoxicity in the mouse lung cancer model but did not synergically inhibit the tumor growth on top of DDP treatment.

## Data Availability

The original contributions presented in the study are included in the article/Supplementary Material, further inquiries can be directed to the corresponding authors.
