# Supplementary material for: An Optimized Two-Herb Chinese Food as Medicine Formula Reduces Cisplatin-Induced Nephrotoxicity in the Treatment of Lung Cancer in Mice
Source: Front Pharmacol. 2022 Mar 8;13:827901. doi: 10.3389/fphar.2022.827901 (PMC8959097; doi:10.3389/fphar.2022.827901)

Supplementary material

HPLC profiles of different components in the herbal and aqueous extracts of Gan and Ju. Details of chromatographic detection method and experimental procedures are given in materials and methods.

Figure S1 HPLC profiles of standard GA (HPLC trace a) and herbal Gan (HPLC trace b,c,d).


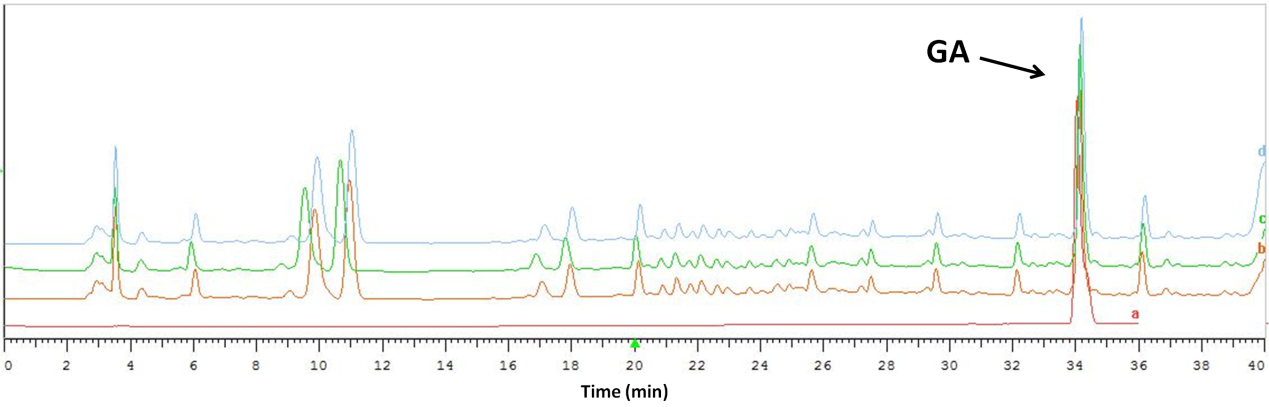


Figure S2 HPLC profiles of standard 3-CQA (HPLC trace a),3,5-diCQA (HPLC trace b),LUS (HPLC trace c)and herbal Ju (HPLC trace d,e,f).


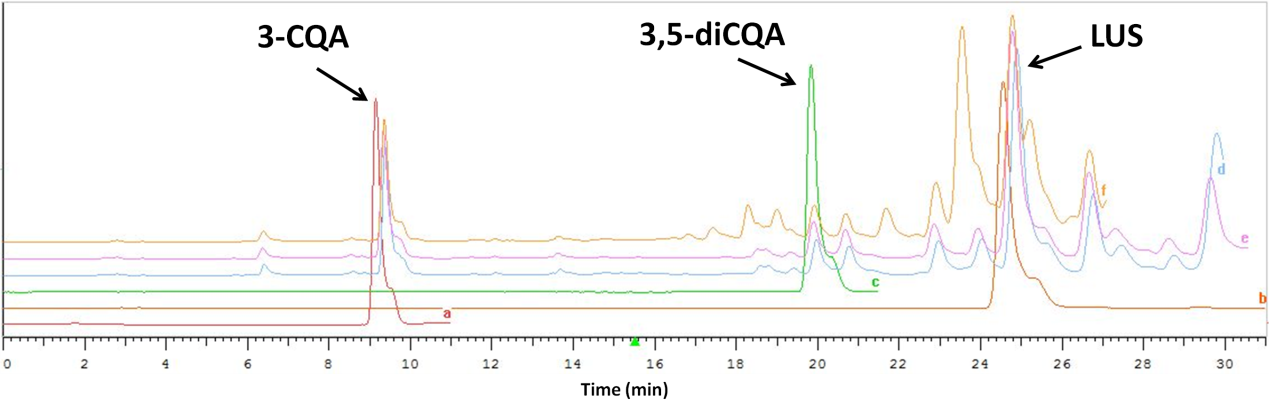


Figure S3 HPLC profiles of standard GA (HPLC trace a) and aqueous extracts of Gan (HPLC trace b,c,d).


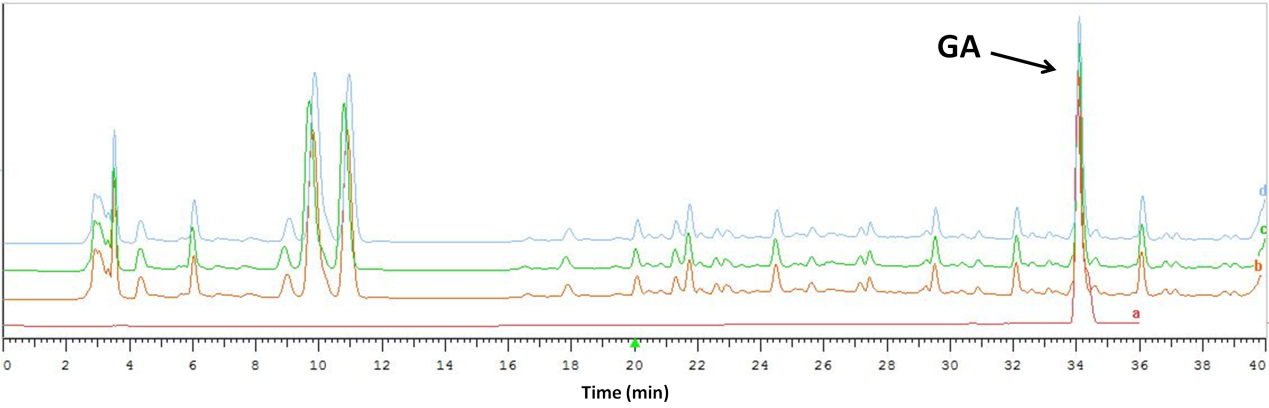


Figure S4 HPLC profiles of standard 3-CQA (HPLC trace a),3,5-diCQA (HPLC trace b),LUS (HPLC trace c)and aqueous extracts of Ju (HPLC trace d,e,f).


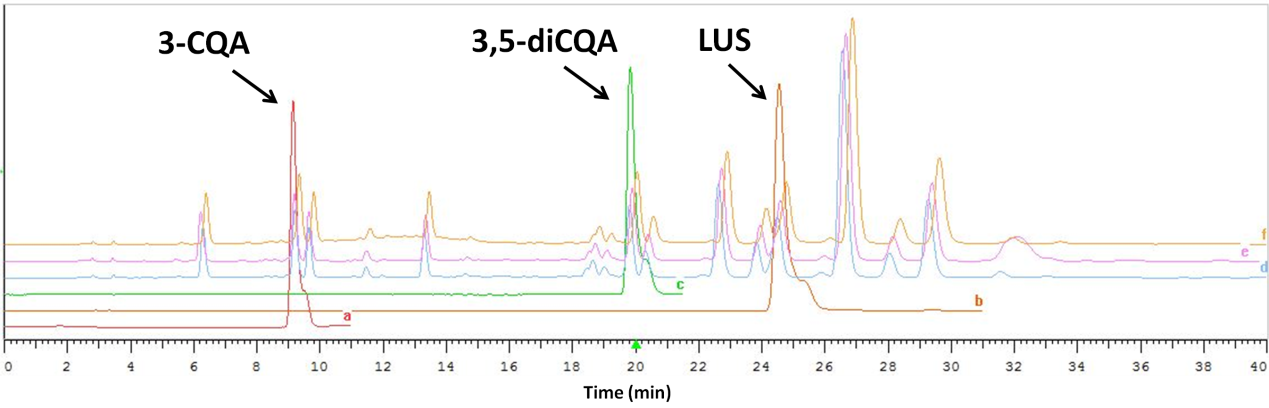

Supplement: Supplementary file 1 [file DataSheet1.docx]
